# Mechano-electrical-fluid interaction left-ventricle model for numerical evaluation of aortic valve hemodynamics

**DOI:** 10.3389/fbioe.2025.1713023

**Published:** 2026-01-07

**Authors:** Nikita Pil, Alex G. Kuchumov, Fulufhelo Nemavhola, Thanyani Pandelani, Truong Sang Ha

**Affiliations:** 1 Biofluids Laboratory, Perm National Research Polytechnic University, Perm, Russia; 2 Department of Computational Mathematics, Mechanics and Biomechanics, Perm National Research Polytechnic University, Perm, Russia; 3 Research Center for Genetics and Life Sciences, Sirius University of Science and Technology, Sirius, Russia; 4 Department of Mechanical Engineering, Faculty of Engineering and the Built Environment, Durban University of Technology, Durban, South Africa; 5 Department of Mechanical, Bioresources and Biomedical Engineering, School of Engineering, College of Science, Engineering and Technology, University of South Africa, Pretoria, South Africa; 6 Department of Mechanical Engineering, Le Quy Don Technical University, Hanoi, Vietnam

**Keywords:** aortic valve, left ventricle, electrophysiology, fluid-structure interaction (FSI), mechano-electrical-fluid interaction model

## Abstract

**Background and Objective:**

Aortic valve simulation has a crucial meaning for clinical applications like the prediction of transcatheter aortic valve implantation or the Ozaki procedure. One of the main aspects is the inflow boundary condition because it has a strong effect on hemodynamic flow simulation results. Most researchers adopt a 2-D profile derived from ultrasound measurements for 3-D fluid-structure interaction simulations that do not take into account several physiological effects.

**Methods:**

A model including left ventricle contraction and blood flow in the aorta segment with aortic valve leaflets was developed. A mechano-electrical-fluidic interaction model of the left ventricle was developed to assess a 3-D profile of blood passing to the aortic valve. The effect of complex fiber architecture in the left ventricle geometry model was taken into account. After that, this profile was set as an inlet in the aorta segments to perform 2-way FSI blood flow for numerical evaluation of aortic valve hemodynamics.

**Results:**

It was shown that during the cardiac cycle, the left ventricle’s electric potential varies between −80 mV and 20 mV. At the systolic peak, the maximum deformations of the left ventricle range from 38% to 60%. The trajectories of the left ventricle apex and torsion angle were derived. The displacement of the myocardial tissue does not differ significantly among the cases, ranging from 15 to 20 mm, with the greatest shift occurring in the opposite direction. Flow velocities were up to 1.8 m·s^−1^ at the moment of full opening of the aortic valve leaflets. Additionally, the influence of the left ventricle’s shape and size on the left ventricle outflow velocity vector field and the aortic valve leaflets’ behavior was analyzed.

**Conclusion:**

The findings suggest that ventricular geometry significantly influences the stress distribution in the aortic valve leaflets and the flow velocities, consistent with previous computational studies. Understanding these relationships is crucial for predicting valve performance and identifying potential areas of high stress that may contribute to valvular pathologies such as calcification and leaflet fatigue.

## 1 Introduction

Computational models of the heart and its chambers can be divided into several groups by simulation complexity and effects that these models are able to capture (Lopez-Perez et al., 2015; Lopez-Perez et al., 2019).

These models include fluid–structure interaction (FSI) (taking into account the impact of blood flow on cardiac tissue deformation effect) (Mao et al., 2017), mechano–electrical interaction (MEI) (adopting the mechanical contraction of heart induced by electrical excitation) (Verzicco, 2022), and mechano-electrical-fluid interaction (MEFI) (which takes into account all three effects: hemodynamics, electrical potential propagation and mechanical response) (Arefin and Morsi, 2014).

In silico MEFI models provide more accurate and realistic results to be adopted in clinical applications for proper patient-specific decision-making. These models are able to connect the effects of disease progression such as arrythmia or myocardial infarction with blood flow alterations and soft tissue response during a heart cycle contraction.

Only a few models present MEFI computational frameworks applying either Robin–Neumann interface conditions to reduce FSI computational costs (Bucelli et al., 2023a; Bucelli et al., 2023b) or immersed boundary method (where the heart is embedded in a larger fluid computational domain enhanced by low-dimensional models (0D models) representing systemic circulation (Viola et al., 2023). Several MEFI models utilize only 1-way FSI approach with mechanical deformation not feeding back into the electrical activity (Watanabe et al., 2004; Vigmond et al., 2008) which limits their ability to totally mimic heart function. Despite these limitations, the work represents a significant advancement by unifying multiple disciplines and highlighting the value of interdisciplinary approaches in tackling complex cardiovascular simulations. More complex comprehensive and multiphysics mathematical models suitable for surgical planning and clinically relevant decision-making have been recently proposed (Quarteroni et al., 2023; Gonzalo et al., 2024; Brown et al., 2025).

Good comprehensive reviews of MEFI cardiac models have recently published (Torre et al., 2023; Xu and Wang, 2025). It should be noted that these models have not been used for clinical applications to analyze and predict aortic valve function after surgical treatment of aortic stenosis.

Computational fluid dynamics (CFD) (Kuchumov et al., 2015; Kuchumov et al., 2021; Youssefi et al., 2017; Hellmeier et al., 2018; Hoeijmakers et al., 2019; Kuchumov et al., 2020; Hashemifard et al., 2024; Liu et al., 2024) and fluid-structure interaction (FSI) (Marom, 2015; Sodhani et al., 2018; Spühler et al., 2018; Kuchumov et al., 2021; Abbas et al., 2022; Le et al., 2022; Morany et al., 2023; Khairulin et al., 2024) are widely used for assessment of clinical parameters. Nevertheless, this approach is still doubted to be a reliable tool for real decision-support applications. Thus, there are several challenges in developing accurate computational models to guide surgical interventions. These challenges include the choice of the correct soft tissue model describing the mechanical behavior of aortic valve leaflets (Pil et al., 2023), design of the complex geometry of aortic valve cusps (Vassilevski et al., 2021; Pase et al., 2023; Macé et al., 2024), and the turbulence model (Becsek et al., 2020; Manchester et al., 2021; Perinajová et al., 2021; Martínez et al., 2023).

However, the most debatable issue is inflow boundary condition settings, because they have a strong effect on hemodynamic flow simulation results. Steady-state velocity applied as inlet boundary conditions (BC) does not correspond to reality and should be avoided (Smadi et al., 2009; Armour et al., 2021; Hoeijmakers et al., 2022; Nebogatikov and Pichkhidze, 2024). Unsteady time-dependent velocity of the plug flow is usually applied as the inflow boundary condition (Amindari et al., 2017; 2021; Gilmanov et al., 2018; Gilmanov et al., 2019; Chi et al., 2022). This means that the normal velocity component derived from clinical data (for example, Doppler measurements) is taken into account (González-García et al., 2024; Riccardi et al., 2024). This approach has a serious drawback: it does not include the effects of helicity and vorticity, which occur in the result of left ventricle contraction (Pierrakos and Vlachos, 2006; Schäfer et al., 2020). Moreover, this kind of time-dependent velocity profile mostly corresponds to a healthy individual and cannot be used in patient-specific simulations. 0-D (Cai et al., 2021) and 1-D (Simakov, 2019) derived profiles are also adopted, but they also have a similar disadvantage. According to 4D MRI data (Lorenz et al., 2014; Ramaekers et al., 2023; Calò et al., 2024), blood flow from the left ventricle is displaced relative to the central axis of the aortic valve and has a complex vortex structure and helicity (Mehmood et al., 2024).

Pressure-based BC applied at the inlet can serve as an alternative to velocity-based ones (Chen et al., 2022; Yin et al., 2024), but a proper convergence study should be performed. Moreover, this approach has limitations as it does not use clinically measured parameters such as cardiac output or heart rate.

A few investigations consider the velocity profile set at the inlet BC (Cao and Sucosky, 2015; Armour et al., 2021) to assess the impact of the plug and the patient-specific velocity profile. However, using the computational fluid dynamics (CFD) approach to describe the hemodynamics of the aortic valve is a limitation of their models. Doost et al. (2016) review of approaches to left ventricular modeling indicates that most studies rely on CFD methods with prescribed wall motion (Obermeier et al., 2022) derived from medical imaging analysis. Xu and Kenjereš (2021) proposed an alternative approach utilizing the radial basis function (RBF) method for mesh morphing to describe left ventricular deformation.

Using only CFD is limited because it does not consider flexible wall deformation by blood flow and *vice versa*. The fluid-structure interaction (FSI) approach can simulate aortic valve leaflets opening/closing and evaluate stress-strain distribution in the aorta. It provides a more accurate description of cases where flow–structure interaction determines the dynamics. For example, Arefin’s models of the left ventricle (Arefin and Morsi, 2014) without interaction with the aortic valve can be mentioned. In contrast, Mao et al. (2017), Terahara et al. (2020), Xu et al. (2021), Govindarajan et al. (2022), and Le et al. (2022) focused on simulating the interaction between the left ventricle and the aortic valve, enabling more accurate assessment of hemodynamic parameters. However, these approaches often overlook electrophysiological effects, which play a crucial role in myocardial mechanics.

Electrophysiological processes, including the propagation of electrical excitation through the myocardium and the activation of myocardial contraction, are comprehensively described by Bakir et al. (2018) and Willems et al. (2024). These works illustrate how the intricate myocardial fiber architecture and the mechanisms of active and passive contraction significantly influence intraventricular flow patterns. Thus, despite the substantial body of research in this field, the development of a comprehensive model that simultaneously accounts for left ventricle–aortic valve interaction within an FSI framework and explicitly models electrophysiological processes remains an open challenge.

In this study, we aim to develop a comprehensive, patient-specific three-dimensional (3D) model that encompasses both the left ventricle (LV) and the aortic segment with the valve leaflets. The velocity vector field (VVF) at the inlet to the aortic segment is derived from the MEFI model of LV contraction, enabling the use of boundary conditions that realistically capture the interaction of myocardial mechanical properties and electrophysiological processes. First, we seek to demonstrate that this methodology can coherently integrate the mechanical and electrophysiological aspects of the LV—encompassing active myocardial contraction, anisotropic tissue properties, and leaflet–flow interaction—into a detailed FSI-based framework for the aortic valve.

Second, we aim to illustrate how various LV morphologies (for example, changes in wall thickness or chamber volume characteristic of dilation or hypertrophy) can significantly affect both the velocity profile near the aortic valve and the distribution of wall shear stress (WSS) on the leaflets. Moreover, by explicitly accounting for the electrophysiological component, we can investigate how different conduction or rhythm alterations influence contraction synchrony and, consequently, aortic valve hemodynamics.

The manuscript is arranged as follows. Section 2 describes LV geometries and the models adopted in the study. Effects of LV dimensions on simulation results are considered. Also, this section contains information about the mesh, material models, and FSI settings. Section 3 presents the results of simulations. In Section 4, the obtained results are discussed and validated with known data. Finally, the study’s limitations are discussed in Section 5.

## 2 Methods

We proposed a complex model to compute blood flow through the aortic valve. The model includes the left ventricle and the aorta segment with the aortic valve leaflets. The left ventricle geometric model is designed based on ultrasound measurements and takes into account the complex fiber architecture. Moreover, an electromechanical model coupled with FSI can describe the systole–diastole physiological behavior. This model is necessary to obtain a time-dependent velocity field vector, which can serve as a boundary condition at the inlet for the aorta segment. Additionally, the influence of the left ventricle’s shape and size on the left ventricle outflow velocity vector field is analyzed.

**FIGURE 1:**
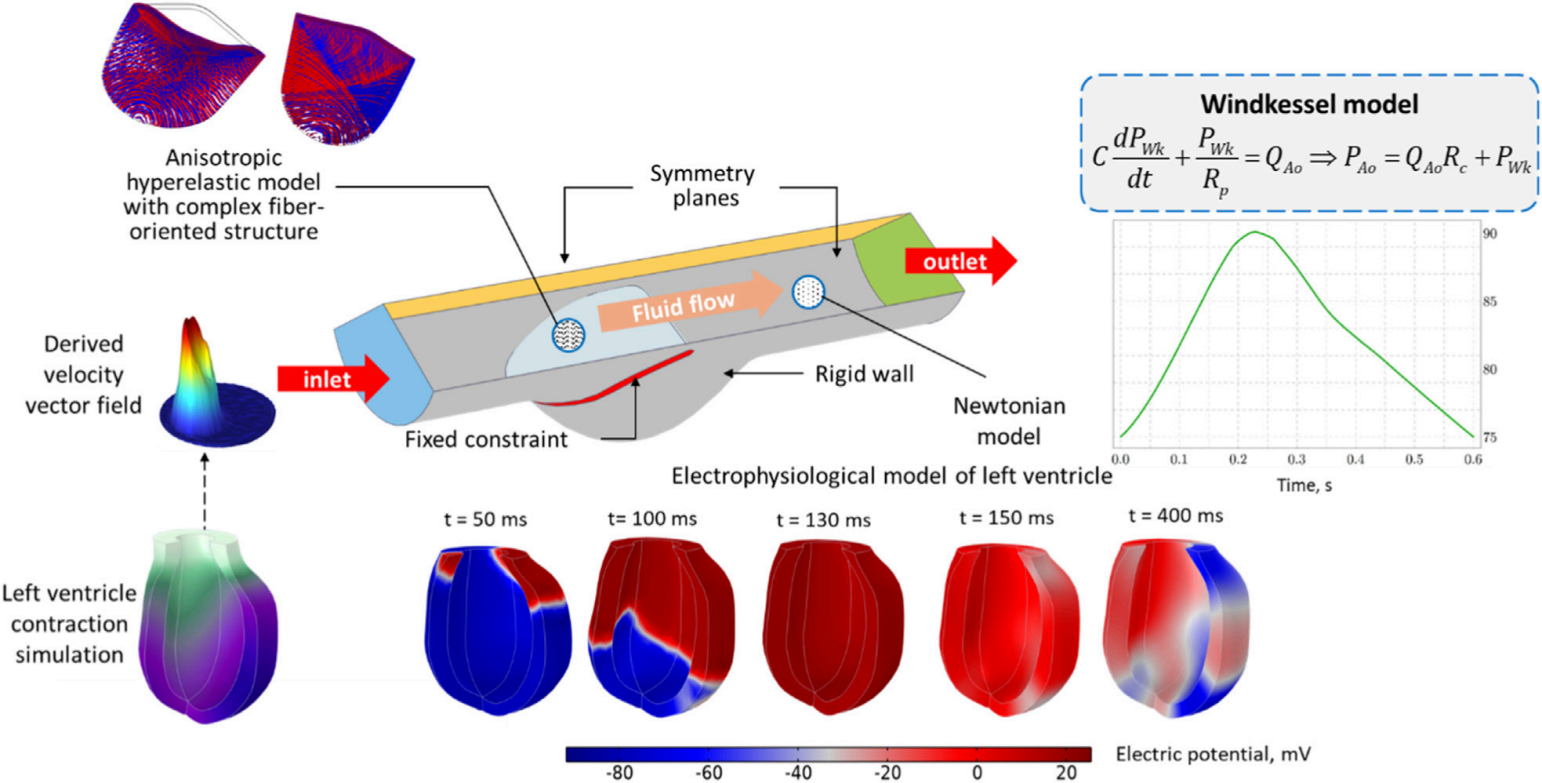
Design of study (MEFI left ventricle (LV) model and aorta segment with aortic valve leaflets model. At first, MEFI model is realized to evaluate velocity vector field at LV outlet. After that, this 3-D unsteady velocity profile is set as boundary condition at inlet of aortic root with three leaflets (with complex fiber-oriented structure). Blood is considered as Newtonian fluid. Windkessel model is set at outlet).

The model also accounts for the aortic valve leaflets’ fiber orientation. An anisotropic hyperelastic material model was used to describe the leaflets’ dynamic behavior. It was previously shown to be more precise than an isotropic hyperelastic model. Windkessel model pressure dependence was applied at the outlet of the aorta segment. Modeling of the blood flow in the aorta segments and simulation of opening/closing aortic valve leaflets are solved by adopting the FSI approach. The design of study is shown in Figure 1.

### 2.1 Left ventricle performance simulation

#### 2.1.1 Data acquisition and image processing

CT scans of the left ventricle and aorta of a healthy 39-year-old male volunteer (height = 179 cm, weight = 89 kg) were acquired using a Siemens SOMATOM CT scanner. Moreover, ultrasound imaging using Hitachi Aloka Arietta S70 scanner was performed to get patient data during systole and diastole at MEDSI Clinical Center (Perm, Russia). The study was approved by the Ethical Committee (protocol No. 22 on 06 May of 2024) and written informed consent was obtained.

#### 2.1.2 Left ventricle geometric model post-processing and parameterization

There are many approaches to geometric modeling of the left ventricle and other organs. Computed tomography is commonly used, followed by segmentation and creation of finite elements for the model (Obermeier et al., 2022; Xu et al., 2022). This allows for high-precision anatomy of a specific patient, but it depends on image quality and increases computational complexity, which limits the ability to create large model databases. An alternative approach uses synthetic geometries (Buoso et al., 2021; Babaei et al., 2022).

**FIGURE 2:**
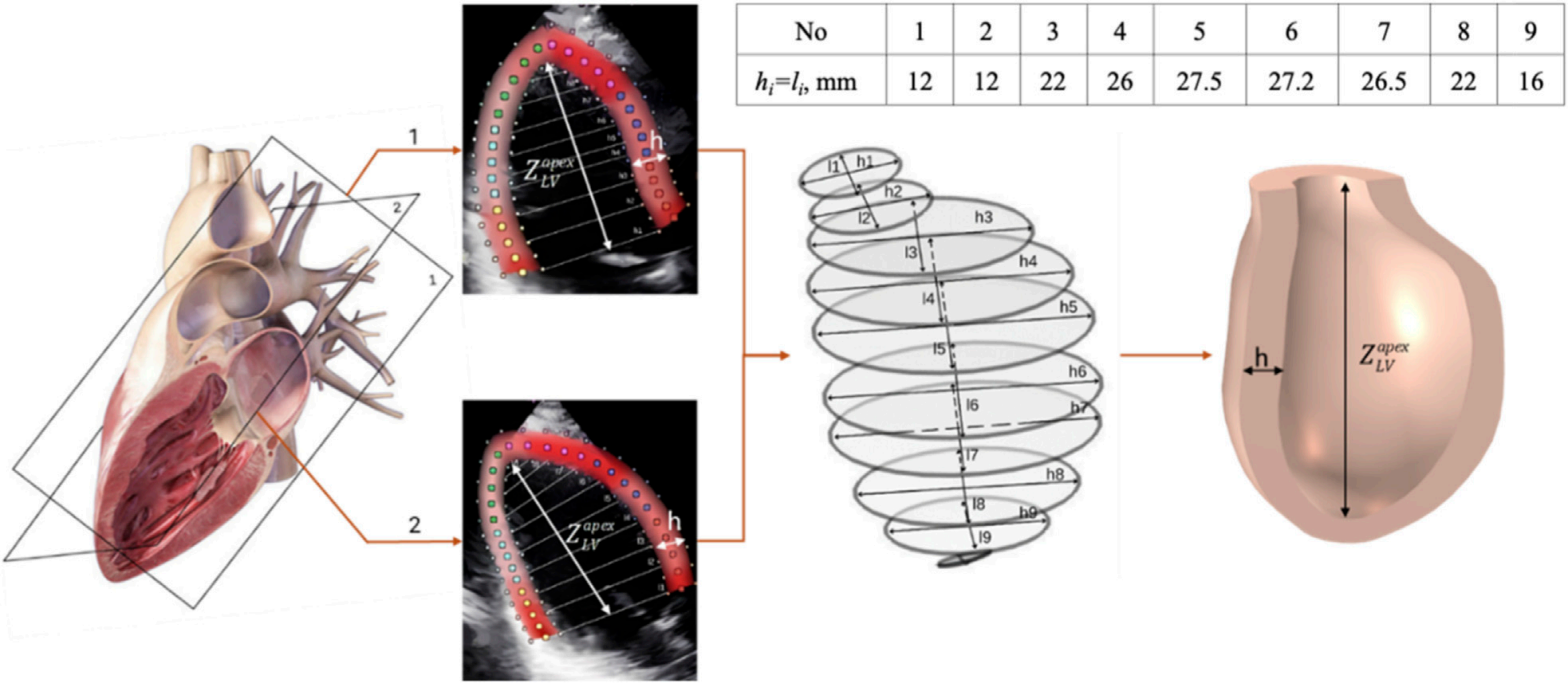
Left ventricle geometry. The LV is designed by number of elliptical cross-sections from apex to base. The endocardium and epicardium define the wall thickness map.

Shapes and sizes are parameterized by curves with limited ranges of coefficients, allowing for the rapid creation of diverse models and systematic evaluation of the impact of size variations on physiological processes. This approach is effective for large-scale studies and complements patient-specific modeling by covering a wider range of anatomical variability and supporting statistical analysis and machine learning (Bian et al., 2025; Krones et al., 2025). Elliptical cross-sections along the central axis define the endocardial and epicardial surfaces in echocardiography images, as well as the spatially varying wall thickness, and allow control of local thickening and global shape changes are shown in Figure 2. The resulting solid model encompasses the cavity and serves as the computational domain to model blood flow.

The endocardium and epicardium are segmented by orthogonal apical projections of the LV. Along the apex-to-base axis, nine orthogonal planes are located; in each plane, the LV contour is approximated by an ellipse:

$$\frac{{\left(x-x_{c}\left(s_{i}\right)\right)}^{2}}{a_{endo}{\left(s_{i}\right)}^{2}}+\frac{{\left(y-y_{c}\left(s_{i}\right)\right)}^{2}}{b_{endo}{\left(s_{i}\right)}^{2}}=1,$$ (1)

where s is the parameter along the left-ventricle centerline; i is the cross-section index along the axis, $x_{c}\left(s_{i}\right),y_{c}\left(s_{i}\right)$ are the coordinates of the ellipse center in the section at parameter s , $a_{endo}\left(s_{i}\right),b_{endo}\left(s_{i}\right)$ are the semi-axes of the endocardial ellipse in the section at parameter s, with a along the major axis and b along the minor axis.

The epicardium is defined similarly by elliptical approximations of the epicardial contours in the same planes, which allows the thickness map to be calculated as the normal distance between the epicardial and endocardial ellipses at each point:

$$t\left(\theta ,s\right)=\left(X_{epi}\left(\theta ,s\right)-X_{endo}\left(\theta ,s\right)\right)\cdot n_{endo}\left(\theta ,s\right),$$ (2)

where $t\left(\theta ,s\right)$ is the wall thickness; θ is the circumferential angle in the cross-sectional plane, $X_{endo}$ and $X_{epi}$ are the position vectors of the endocardial and epicardial points on the ellipse at angle θ and parameter s , respectively, $n_{endo}$ is the unit outward normal to the endocardium at that point.

Based on Equations 1, 2, five synthetic LV geometries were created. The synthetic geometries were characterized by key geometric parameters (ranging from normal anatomy to different patterns of myocardial remodeling). In the final step, each model’s myocardial walls were equipped with a distributed fiber system that imposes the LV’s anisotropic mechanical properties.

Our model focuses on simulating systolic ejection and evaluating the impact of different flow fields on the aortic valve. As a result, we simplified the geometry to a single opening in the outflow tract. Fully coupling FSI with the mitral and aortic valves would require many more degrees of freedom, a more complex contact algorithm, and much higher computational costs. Diastolic filling is not modeled. We start with the end-diastolic configuration, considered as an effective pre-stressed initial state. The simulation then continues through isovolumic contraction and ejection until the aortic valve closes. This volume limits the interpretation of the left ventricle results to the systolic phase.

We developed five distinct geometric models of the LV, each focusing on one of the key geometric parameters commonly encountered in various forms of myocardial remodeling. In the current study, LVOT was not extracted from the images. It was modeled parametrically as a short axial section between the basal endocardial ellipse and the valve plane. The geometry was constructed as a smooth transition with a continuous normal. Subvalvular structures were not taken into account. The anterior leaflet of the mitral valve was not modeled. The LVOT cross-section followed the basal ellipse and gradually aligned with the cross-section at the valve annulus. The goal was to obtain a consistent jet extension to the valve plane without the influence of local irregularities. In the first model, the LV walls are thickened, illustrating concentric hypertrophy, in which wall thickness increases substantially while the ventricle’s internal volume remains nearly unchanged. Such an adaptation is often observed under elevated afterload (e.g., arterial hypertension). The second model highlights a more pronounced change in the “height” or longitudinal dimension of the LV, reminiscent of eccentric hypertrophy or other remodeling types that produce an elongated ventricular shape. The third model modifies the radius of the LV outflow cross-section, directly influencing the velocity profile and pressure during the systole; even minor changes in this area can noticeably affect aortic valve hemodynamics. The fourth model alters the inclination angle of the outflow tract, thereby shifting its spatial orientation in a way that induces significant flow disturbances and uneven stress distributions on the valve leaflets. Finally, the fifth model applies multiple geometric modifications simultaneously, creating a more comprehensive remodeling scenario that reflects the multiparametric pathological states commonly seen in chronic cardiac conditions. The electrophysiological model was used to compute these models of the left ventricle, the data for which are presented in Table 1. The geometries are consistent in volume, length of the long axis, and radius of the LVOT.

**TABLE 1:** Clinical data for LV models.

Parameters	Case 1	Case 2	Case 3	Case 4	Case 5
Volume, mm^3^	36.2	36	31.7	45.4	63.1
EDV, mm^3^	117.3	122.5	83.2	117,5	157.5
EF, %	69.1	70.6	61.9	61.3	60
SV, mm^3^	81.1	86.5	51,5	72,1	94.4
Long-axis length, mm	74.2	74.8	77.9	84.6	86.1
LVID, mm	54.4	54.4	50	54.4	62.4
Mass, g	144	223.8	140.4	144.6	164.1
PWTmax, mm	7.3	11	10.2	7.6	9.4
PWTmin, mm	5	4.8	5.3	5.2	4.2
PWTavg, mm	6.2	8.4	8.7	6.6	7.7
RVWTmax, mm	6.5	10.1	9.4	6.8	8.5
RVWTmin, mm	3.3	4.8	4.4	3.5	4
RVWTavg, mm	5	7.1	6.9	5.2	5.4
ISTmax, mm	8.3	12.1	11.4	8.7	9.1
ISTmin, mm	5.3	4.8	4.7	5.6	4.3
RWTSavg, mm	6.9	9.3	8.6	7.1	6.5
RWTmax,mm	7.3	11	10.1	7.5	8.3
RWTmin,mm	5.1	4.8	4.5	5.3	4.4
RWTavg,mm	6.3	8.6	8.2	6.4	6.1
WTmax, mm	8.4	12.1	11.7	8.6	8.8
WTmin, mm	3.3	4.8	4.5	3.4	4
WTavg, mm	6.2	9.3	9.1	6.7	6.5

Abbreviations: D, diastole; S, systole; EDV, end diastolic volume; EF, ejection fraction; SV, stroke volume; LVID, left ventricular internal dimension; PWT, posterior wall thickness; RVWT, right ventricular wall thickness; IST, interventricular septal thickness; RWT, relative wall thickness; WT, total wall thickness.

#### 2.1.3 MEFI model

##### 2.1.3.1 Electrophysiological model

The myocardial microstructure is defined by the orientations of fibers, sheets, and normal-to-sheet axes (Bakir et al., 2018).

$$\theta_{epi}=\theta_{epi}{\;}^{\max}\left(1-\frac{Z}{Z_{LV}^{apex}}\right),\theta_{end}=\theta_{end}{\;}^{\max}\left(1-\frac{Z}{Z_{LV}^{apex}}\right)$$ (3)

$$\theta =\beta \theta_{end}+\left(1-\beta \right)\theta_{epi},\beta =\frac{D_{epi}}{D_{epi}+D_{end}}$$ (4)

where $\theta_{epi}$ and $\theta_{end}$ are fiber orientations on epicardium and endocardium, $\theta_{epi}{\;}^{\max}$ and $\theta_{end}{\;}^{\max}$ are epicardial and endocardial fiber angle at basal plane, $Z_{LV}^{apex}$ is z-coordinate of LV apex, β is a dimensionless wall distance parameter, θ is a fiber orientations in myocardium, $D_{epi}$ and $D_{end}$ is a distance from outer boundaries of epicardium and endocardium.

To simplify the model, several settings and assumptions were adopted. The fibers’ orientation was set to be $\theta_{epi}{\;}^{\max}=-60{}^{\circ}$ relative to the circumferential plane at the epicardium and $\theta_{end}{\;}^{\max}=60{}^{\circ}$ at the endocardium, with a linear transmural variation $\theta \left(\beta \right)$ . The parameter β represents the distance of fibers to the epicardial boundary, where 0 = epicardium and 1 = endocardium. The microstructural sheets were assumed to be perpendicular to both the epicardial and endocardial surfaces. Due to the experimental difficulty of measuring the apical microstructure and to avoid singularities from applying the above principles, the apex region, defined by a 1-cm-diameter cylinder, was assumed to have an isotropic microstructure. Fiber architecture is based on literature data (see Li et al., 2025). Moreover, our approach can take into account any fiber orientation, which can be derived from imaging data. Validation and comparison added to Section 3.3

The propagation of the myocardial action potential *Ф* is described as follows:

$$\chi_{m}C_{m}\frac{\partial \Phi }{\partial t}+\nabla \left(-D\nabla \Phi \right)+\chi_{m}I_{ion}\left(\Phi ,F,r\right)=0,$$ (5)

where $\chi_{m}C_{m}$ acts as the damping coefficient. Here, $\chi_{m}$ is the surface-to-volume ratio, m^−1^, $C_{m}$ is the membrane capacitance, F·m^−1^, $I_{ion}$ is the ionic current, Ф is a myocardial action potential, F is a deformation gradient, r is the internal (recovery) variable.

The conductivity tensor $D=\left(d_{iso}C_{m}\chi_{m}\right)I+\left(d_{ani}C_{m}\chi_{m}\right)a_{0}⊗a_{0}$ includes both isotropic and anisotropic components, which depend on fiber orientation (Göktepe and Kuhl, 2010).

To match the experimental values of electrical potential and activation time in the myocardium, a dimensional transformation of the ionic current is used:

$$I_{ion}=C_{m}\frac{\beta_{\varphi }}{\beta_{t}}\left({\tilde{I}}_{e}+{\tilde{I}}_{m}\right).$$ (6)

The ionic current $I_{ion}$ is the sum of the currents induced by excitation (purely electrical) ${\tilde{I}}_{e}$ and those induced by stretch ${\tilde{I}}_{m}$ . The relationships for the electrical component of the ionic current are derived from the Aliev–Panfilov equation (Nash and Panfilov, 2004). Currents induced by excitation (purely electrical) ${\tilde{I}}_{e}$ can be defined as

$${\tilde{I}}_{e}\left(\varphi ,r\right)=c\varphi \left(\varphi -\alpha \right)\left(\varphi -1\right)+r\varphi ,$$ (7)

where φ is the dimensionless potential, r is the internal (recovery) variable, and α,c are the Aliev–Panfilov parameters. The equation defines the electric reaction current, and the cubic polynomial implements fast positive feedback and rest-excitation bistability: At φ ≈0, the system is stable; when the threshold *α* is exceeded, a rapid rise to a plateau occurs ( φ →1). The coefficient *c* scales the steepness and speed of the ascending phase of the action potential; the parameter *α* determines the response threshold. The term rφ represents a slow “inhibitory” (restorative) current, which increases at large φ and thereby ensures repolarization and the refractory period.

Stretch-induced currents can be computed as

$${\tilde{I}}_{m}\left(F\right)=\theta G_{s}\left(\lambda \left(F\right)-1\right)\left(\varphi -\varphi_{s}\right),$$ (8)

where *λ*(*F*) is the local stretch of the fibers (the function of the deformation gradient *F*), so that *λ*(*F*) – 1 measures the deviation of the sarcomere length from the “resting” length. The factor *θ* is the time window of active response generation. *G*_*s*_ is the gain of the length-dependent activation. The factor *ϕ*_s_ introduces a threshold for the electrical state. The mechano-electrical contribution is included only after reaching the electrical activation *ϕ*_*s*_, which reconciles electromechanics with excitation-contraction physiology. This linear-affine form in *λ* compactly implements the Frank–Starling effect: When the fiber is lengthened (*λ* > 1), the activating contribution is enhanced, and when it is shortened, it is weakened.

The kinetics of the recovery variable is written as follows

$$\frac{\partial r}{\partial \tau }=\left(\gamma +\frac{\mu_{1}}{\mu_{2}+\varphi }r\right)\left(-r-c\varphi \left(\varphi -b-1\right)\right),$$ (9)

The first factor $\gamma +\frac{\mu_{1}}{\mu_{2}+\varphi }r$ regulates the rate of evolution of *r*. At high *ϕ* and large *r*, recovery is accelerated, which reproduces the phenomena of restitution (dependence of the duration of the active potential on the prehistory). The second factor $-r-c\varphi \left(\varphi -b-1\right)$ specifies the quasi-stationary “goal” toward which *r* tends. During the plateau (*ϕ* is large), *r* is pressed to small values (inhibition is released); during repolarization (*ϕ* falls), the sign changes, and *r* returns to the base level, restoring excitability. The parameter *b* shifts the position of the “break” along *ϕ* and controls the duration of the plateau; *μ*_1_, *μ*_2_ and *γ* specify the shape of the restitution curves and the temporal asymmetry: «fast excitation, slow recovery».

The dynamic action of active stress $\sigma_{a}$ can be expressed as

$$\frac{\partial \sigma_{a}}{\partial t}=\varepsilon \left(\Phi \right)\left[k\left(\Phi -\Phi_{r}\right)-\sigma_{a}\right],$$ (10)

where $\varepsilon \left(\Phi \right)$ is the delay function (Göktepe and Kuhl, 2010), *t* is the time, *k* is a parameter defining the maximum stress limit, Φ is myocardial action potential, $\Phi_{r}$ is the resting potential.

Delay function takes on the form:

$$\varepsilon \left(\Phi \right)=\varepsilon_{0}+\left(\varepsilon_{0}+\varepsilon_{1}\right)\exp\left(-\exp\left(-\zeta \left(\Phi -\Phi_{t}\right)\right)\right),$$ (11)

where $\varepsilon_{0}$ and $\varepsilon_{1}$ are the contraction rate constants, Φ is myocardial action potential, $\Phi_{t}$ is potential phase shift, and ζ is the transition rate.

Myocardial action potential is written as follows

$$\Phi =\varphi \beta_{\varphi }+\delta_{\varphi },$$ (12)

$${t=\beta }_{t}\tau$$ (13)

$$\beta_{t}=t_{\beta }\cdot \left(1-\tau_{0}\frac{t_{a}-t_{0}}{t_{1}-t_{0}}\right),$$ (14)

$$t_{a}=t_{\alpha }\cdot \left(1-\frac{Z}{Z_{LV}^{apex}}\right)$$ (15)

where parameters $\delta_{\varphi }$ and $\beta_{\varphi }$ are chosen according to the experimental values of the heart’s resting potential, which is −80 mV, and the maximum potential value of 20 mV. The temporal scaling parameter $\beta_{t}$ is considered to be dependent on the activation time $t_{a}$ , with $\tau_{0},t_{0},t_{1},t_{\alpha }$ and $t_{\beta }$ being tuning parameters, ms. During the cardiac cycle, the activation time (the time between depolarization and repolarization) is not constant across the myocardium; regions that depolarize last repolarize first.

The active stresses are added *via* the second Piola–Kirchhoff tensor in various proportions along the tensor in different proportions along fibers $\left(\bar{a}\right)$ and sheets $\left(\bar{s}\right)$ , and normal to sheets $\left(\bar{n}\right)$ (Bakir et al., 2018):

$$\sigma =\sigma_{a}\left[\eta_{1}\left(\bar{a}⊗\bar{a}\right)+\eta_{2}\left(\bar{s}⊗\bar{s}\right)+\eta_{3}\left(\bar{n}⊗\bar{n}\right)\right].$$ (16)

Here, the coefficients $\eta_{i},i=\bar{1,3}$ describe the contribution along the anisotropic directions.

The parameters were taken from published sources with experimental evaluation or validation in similar models (Arefin and Morsi, 2014; Mao et al., 2017; Bakir et al., 2018; Obermeier et al., 2022; Poon et al., 2024). Initial values were set within typical physiological limits. Calibration was performed based on systolic and diastolic pressure, stroke volume, ejection fraction, isovolumic phase timing, and the delay between electrical activation and mechanical response. The final ranges and accepted values are presented in Supplementary Material S1.

The electrophysiological module solved an anisotropic monodomain model. The conductivity tensor is oriented along and normal to the fiber sheet. Conductivity along the fibers is 0.6 mm^2^·ms^−1^. Across the fibers, it is 0.2 mm^2^·ms^−1^. Through the thickness, it is 0.1 mm^2^·ms^−1^. The ratio of propagation velocities is approximately 3:1. The orientation of the fibers varies transmurally along a linear helical angle profile with a smooth apicobasal trend. The reaction terms are specified by a two-variable phenomenological model calibrated by the duration of the systolic action potential. Endocardial activation was initiated by a short stimulus in the septal and apical zones. The pulse duration was 2–3 ms. A zero normal current was set on the epicardium. The time step and grid size were chosen to resolve the excitation front with at least five nodes per wavelength. The connection to mechanics was implemented through active voltage with a fixed electromechanical delay.

Finally, based on the LV volume curve and ejection fraction, we adjusted only a small subset of the most sensitive parameters to accurately reproduce the LV volume time course and ejection fraction. Throughout this process, we ensured that all changes remained within the bounds of physiological plausibility.

##### 2.1.3.2 Left ventricle contraction simulation

The process of modeling the excitation, contraction, and ejection from the left ventricle is divided into three stages. In the first stage, a curvilinear coordinate system is established, which is used to construct the fiber distribution in the myocardium wall of the left ventricle. The second stage involves the excitation-contraction processes, where Equations 3–16 are solved to determine the fields of myocardial action potential propagation and active stresses deforming the left ventricle walls. From the solutions of the second stage, the displacement fields of the inner walls of the left ventricle are obtained to calculate the flow velocities within the inner cavity and at the outlet of the left ventricle. For the third stage, a moving mesh interface is utilized, where the velocities and displacements of the nodes are consistent with the calculations from the second stage.

The myocardial stress-strain relationships can be written as:

$$\rho \frac{\partial^{2}u_{s}}{\partial t^{2}}=\nabla \cdot \sigma_{s},$$ (17)

$$\sigma_{s}=\frac{\partial W}{\partial \varepsilon },$$ (18)

$$\varepsilon =\frac{1}{2}\left(\nabla u_{s}+{\left(\nabla u_{s}\right)}^{T}+\left(\nabla u_{s}\right)\cdot {\left(\nabla u_{s}\right)}^{T}\right)$$ (19)

where ρ is the density, $u_{s}$ is the displacement field, $\sigma_{s}$ is the Cauchy stress tensor, ε represents the Green–Lagrange strain tensor, W is the strain energy density.

The myocardium is modeled using anisotropic hyperelastic model with compressible Neo–Hookean isotropic part and HGO (Holzapfel and Ogden, 2009) for anisotropic contribution (see Equations 20–23). This model can replicate the myocardial response to biaxial stretching with four parameters:

$$W=\psi_{isotropic}+\psi_{fiber}$$ (20)

$$\psi_{isotropic}=\frac{1}{2}\mu \left({\bar{I}}_{1}-1\right)+\mu lnJ+\frac{1}{2}\lambda \ln^{2}J$$ (21)

$$\psi_{fiber}=\frac{k_{1}}{2k_{2}}\sum_{i=1}^{2}\left[\exp\left\{k_{2}{\bar{E}}_{\alpha }^{2}\right\}-1\right],$$ (22)

$${\bar{E}}_{\alpha }=\kappa \left({\bar{I}}_{1}-3\right)+\left(1-3\kappa \right)\left({\bar{I}}_{4\left(\alpha \alpha \right)}-1\right),$$ (23)

where $\psi_{isotropic}$ is isochoric isotropic part of the matrix energy, $\psi_{fiber}$ is isochoric anisotropic part of the fiber-family energy, ${\bar{I}}_{1}$ is first isochoric invariant, μ=0.5 MPa and λ=0.2 MPa is Lame parameters, ${\bar{E}}_{\alpha }$ – effective fiber strain measure for family α , ${\bar{I}}_{4\left(\alpha \alpha \right)}$ is isochoric fiber invariant for the family oriented along α , defined as ${\bar{I}}_{4\left(\alpha \alpha \right)}=a\left(\alpha \right)\cdot \bar{C}\cdot a\left(\alpha \right)$ , where $a\left(\alpha \right)$ is the unit fiber direction in the reference state, $k_{1}=1.685$ kPa and $k_{2}=15.779$ positive material parameters of the anisotropic part, κ is a dispersion parameter of fiber orientations with 0 ≤ κ ≤ 1/3, $C_{01}$ and $C_{10}$ are positive parameters of the isochoric isotropic part, J is the relative volume change.

A curvilinear coordinate system (Equations 24–28) is defined by solving the Laplace equation to determine the fiber orientation directions:

$$\nabla \cdot \left(\nabla U\right)=0,$$ (24)

$$e=\frac{-\nabla U}{\left|-\nabla U\right|},$$ (25)

where U – field function, e – normalized vector field defining $a_{i}\left(\alpha \right)$ :

$$a_{1}=\frac{e}{\left|e\right|},$$ (26)

$$a_{2}=\frac{z-\left(z\cdot a_{1}\right)a_{1}}{\left|z-\left(z\cdot a_{1}\right)a_{1}\right|},$$ (27)

$$a_{3}=a_{1}\times a_{2}.$$ (28)

To integrate the electrical and mechanical models, the active stress σ was incorporated into the second Piola–Kirchhoff stress tensor T (Equation 29) along the fiber, sheet, and normal-to-sheet directions:

$$T=\frac{\partial W}{\partial \varepsilon }+\sigma$$ (29)

##### 2.1.3.3 Left ventricle blood flow simulation

Blood flow is considered an incompressible Newtonian fluid flow with a constant density of 1,060 kg·m^−3^ and dynamic viscosity of 0.004 Pa∙s. The Newtonian blood flow model is adopted here because of relatively high Reynolds numbers and shear rates that make non-Newtonian effects negligible. Recently, Lynch, Nama, and Figueroa (2022) performed a computation with 1 million massless particles injected into the arterial anatomical models and tracked for several cardiac cycles. For the arterial model, only a single bolus was released. Hence, there are not many recirculation zones in that region. Secondly, it was shown that the Newtonian model gives approximately the same values as the Carreau–Yasuda model during systole–diastole phases in patient-specific aorta models. Moreover, we focus here on high-shear-rate regions and general flow patterns. Nevertheless, our approach can adopt non-Newtonian models and turbulence flow pattern analysis. We will devote a future study to analyzing turbulence models to simulate patient-specific vortex flows in aortic coarctation.

The continuity Equation 30 and the Navier–Stokes Equations 31–32 to describe fluid flow are written as follows:

$$\nabla \cdot \upsilon_{f}=0$$ (30)

$$\rho \frac{\partial \upsilon_{f}}{\partial t}+\rho \left(\upsilon_{f}\cdot \nabla \right)\upsilon_{f}=\nabla \cdot \sigma_{f}$$ (31)

$$\sigma_{f}=-pI+\tau$$ (32)

where $\upsilon_{f}$ is the fluid velocity, $\sigma_{f}$ is the stress tensor, ρ is the fluid density, p is the pressure, *I* is the identity tensor, and τ is the viscous stress tensor.

##### 2.1.3.4 Fluid-structure interaction coupling

The FSI interface states that the fluid displacements and the solid domain must be compatible. Tractions at this boundary must be at equilibrium, and the fluid must obey the no-slip condition. The following Equations 33–35 describe these conditions:

$$u_{s}=u_{f},\upsilon_{s}=\upsilon_{f},\sigma_{s}\cdot {\hat{n}}_{s}=\sigma_{f}\cdot {\hat{n}}_{f}.$$ (33)

where **u** is a displacement, ***v*** is a velocity, **σ** is a stress; subscripts s and f denote solid and fluid, respectively. The FSI problem was solved using the ALE–FSI approach in the COMSOL Multiphysics (Comsol Inc., Stockholm, Sweden) software package. In the ALE method, the movement of a solid body is described using a reference coordinate system that can be arbitrarily moved without any relation to the structure’s transformation or fluid movement. As a result, both parts effectively combine, and the description located in the transformation flow becomes possible. We used the Navier–Stokes equation to describe the fluid motion. Then, we determined the total force acting on the solid:

$$f=n\cdot \left\{-pI+\left(\mu \left(\nabla u_{f}+{\left(\nabla u_{f}\right)}^{T}\right)\right)\right\},$$ (34)

where p is pressure, μ is dynamic viscosity, and n is normal vector to the solid body boundary.

The Navier–Stokes equations are solved in the spatial (deformed) frame, whereas the equations of solid mechanics are defined in the material (undeformed) frame, so the force must be transformed:

$$F=f\cdot \frac{d\upsilon }{dV},$$ (35)

where dυ and dV are the mesh element scale factors for the spatial frame and the material frame, respectively.

##### 2.1.3.5 Boundary and initial conditions

Zero velocity and pressure are set at the initial moment of time Open boundary is selected for the fluid domain at the outlet:

$$\sigma_{f}\cdot n=-f_{0}n$$ (36)

where $f_{0}$ is the normal stress, Pa. The no-slip boundary condition is defined as follows:

$$u_{f}\cdot n={u_{f}}_{tr}\cdot n,$$ (37)

where ${u_{f}}_{tr}$ is the translational velocity, **u**_**f**_ is the fluid velocity.

**FIGURE 3:**
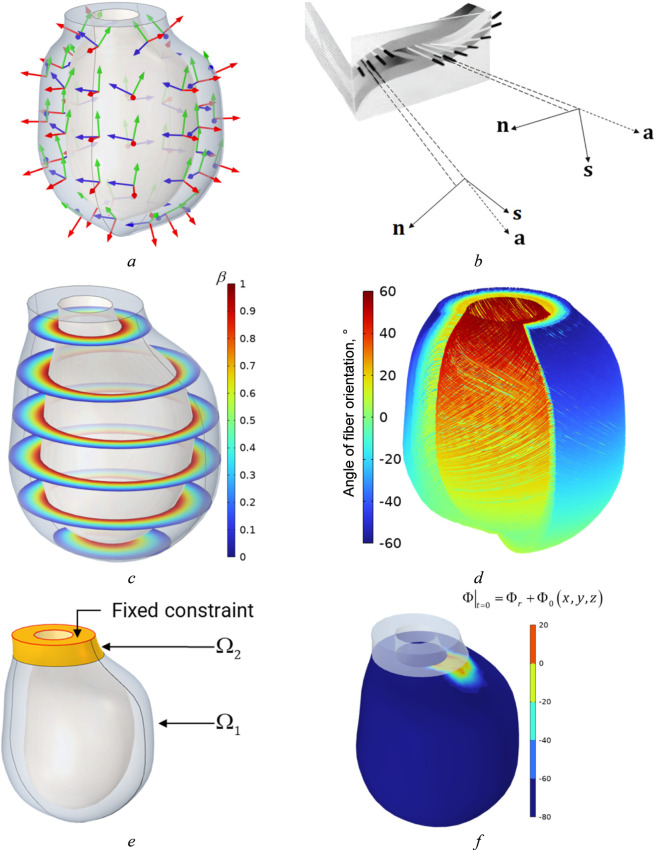
Left ventricle electromechanics: **(a)** 3D model with orientation vectors, **(b)** direction of axes, **(c)** dimensionless parameter of fiber distribution in epicardium and endocardium layers, **(d)** complex fiber architecture in left ventricle, **(e)** boundary condition constraints, **(f)** initial distribution of electrophysiological potential.

The solid body is rigidly fixed in the upper part of the left ventricle (Figure 3e). Recovery variable and active stresses are equal to zero at initial moment of time. The electrophysiological potential at the moment of time is prescribed as:

$${\left.\Phi \right|}_{t=0}=\Phi_{r}+\Phi \left(x,y,z\right)$$ (38)

where $\Phi \left(x,y,z\right)$ is a rectangular domain at the top of LV (Figure 3f). Equations 36–38 set initial and boundary conditions.

### 2.2 Aortic valve performance simulation

#### 2.2.1 Aortic valve geometry design

**FIGURE 4:**
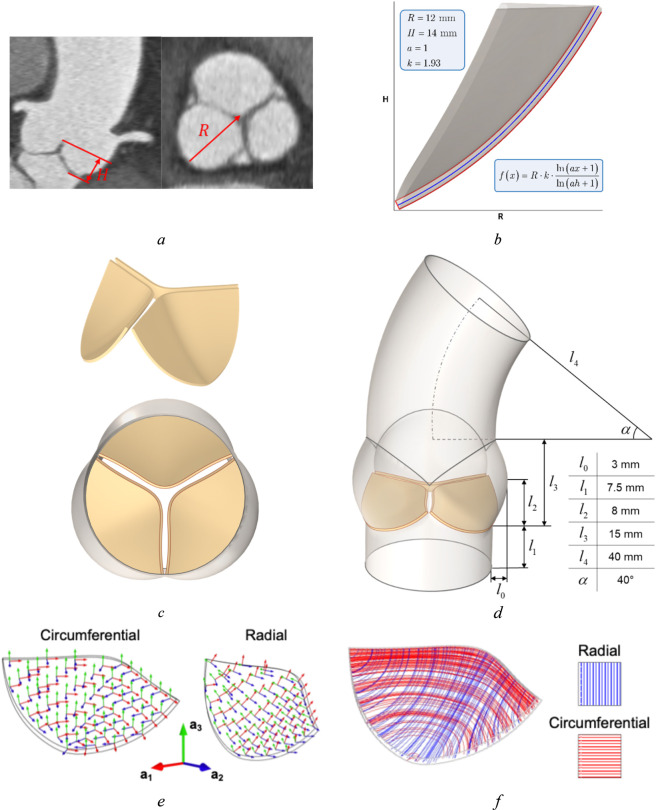
Aortic root with three leaflets model: **(a)** CT image, **(b)** parameterized leaflet geometry described by formula with several parameters (R is a radius, H is a height, *a* and *k* are shape parameters), **(c)** aortic valve geometric model, **(d)** aortic root and aortic valve geometric model with geometric parameters, **(e)** setting up of a curvilinear coordinate system to form the anisotropy of the valves leaflets, **(f)** fiber’s architecture in radial and circumferential directions.

To develop detailed geometries of the aortic valve, it is essential to define key parameters, including the aortic radius and leaflet height (Figure 4a). The shape of each leaflet is controlled by guiding curves (Figure 4b) that are mathematically described using logarithmic functions, thereby ensuring accurate representation of the variability in leaflet morphology. The construction of an idealized geometric model begins by dividing the aortic root into three equal sectors, each corresponding to one of the valve leaflets. A plane perpendicular to the radius of one sector is then established to define the generating curve.

By systematically rotating this plane and adjusting the parameters within the function equations, various geometries of aortic valve leaflets can be generated, allowing for the creation of a diverse range of models (Figure 4c). In summary, the workflow involves obtaining a segmented 3D model of the aorta (Figure 4d), followed by surface smoothing and file conversion, which then supports the subsequent geometric modeling of the aortic valve. This method ensures that both the structural characteristics of the aorta and the specific geometric details of the valve leaflets are precisely captured in the final computational models.

#### 2.2.2 Aortic valve FSI model

The blood properties in the aortic valve model are kept constant. Blood is modeled as a Newtonian fluid with constant density 1,060 kg/m^3^ and viscosity 0.0035 Pa⋅s. The blood flow is governed by the Navier–Stokes equations, similarly to (Equations 30–32).

The mechanical response of the aortic valve leaflets is modeled using the same solid mechanics formulation as for the left ventricle (Equations 17–19). The aortic valve leaflets have a distinct architecture of collagen fibers predominantly located in the radial and circumferential directions (Figures 4e,f). The presence of fibers leads to anisotropic behavior of the leaflets. Modified HGO model is used to describe anisotropic contributions (Equations 39, 40):

$$W=C_{10}\left\{\exp\left[{С}_{01}\left({\bar{I}}_{1}-3\right)\right]-1\right\}+\frac{k_{1}}{2k_{2}}\sum_{i=1}^{2}\left[\exp\left\{k_{2}{\bar{E}}_{\alpha }^{2}\right\}-1\right]$$ (39)

$${\bar{E}}_{\alpha }=\kappa \left({\bar{I}}_{1}-3\right)+\left(1-3\kappa \right)\left({\bar{I}}_{4\left(\alpha \alpha \right)}-1\right).$$ (40)

Here, we adopt $C_{01}=30.03\text{ kPa}$ , $C_{10}=3.47$ , $k_{1}=74.5\text{ kPa}$ , $k_{2}=63.19$ , and κ=0.2 are the material parameters (Mao et al., 2016). The aortic valve leaflets are rigidly fixed along their attachment to the aorta.

The flow velocity is set to zero and the pressure is prescribed as 80 mmHg at the initial moment of time. The inlet velocity field is imposed from the left ventricle model:

$$v_{in}=v_{LVOT},$$ (41)

where $v_{LVOT}$ – velocity vector field from left ventricle outflow. The pressure is determined at the outlet boundary using a two-element Windkessel model:

$$C\frac{dP_{Wk}}{dt}+\frac{P_{Wk}}{R_{p}}=Q_{Ao},$$ (42)

$$P_{Ao}\left(t\right)=Q_{Ao}R_{c}+P_{Wk},$$ (43)

$$p_{out}=P_{Ao}\left(t\right),$$ (44)

where $Q_{Ao}$ is the flow rate in the aorta, mL·s^−1^; $R_{c}$ is the characteristic resistance, mmHg·mL^−1^·s; $R_{p}$ is the peripheral resistance, mmHg·mL^−1^·s; and C is arterial compliance, mm Hg^−1^·mL. $P_{Ao}\left(t\right)$ and $P_{Wk}\left(t\right)$ represent the aortic pressure and pressure stored within the Windkessel model at time *t*, respectively. The first-order differential equation was solved numerically using fourth-order Runge–Kutta. At the current time step, we computed $P_{Ao}\left(t\right)$ using the value of $P_{Wk}\left(t\right)$ defined by the Runge–Kutta solution at the same time step. The model parameters are C=3.128 mmHg^−1^·mL, $R_{p}=$ 0.6652 mmHg·mL^−1^·s, and $R_{c}$ = 0.0914 mmHg·mL^−1^·s. The values for diastolic and systolic pressures are taken as 80 mmHg and 120 mmHg, respectively. Equations 41–44 describe boundary conditions for the fluid domain.

**FIGURE 5:**
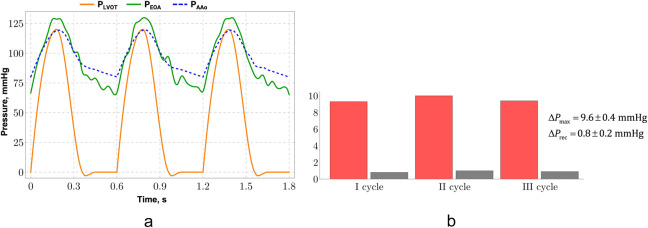
Aortic root with three leaflets model: **(a)** pressure vs. time dependence, **(b)** maximum and relative pressure gradients.

When determining numerical values for the material constants, we relied on studies that performed mechanical testing of aortic walls, valve leaflets, or similar fibrous tissues (Sacks and Yoganathan, 2007; Labrosse et al., 2013; Martin and Sun, 2014). Although there is often no universally fixed set of constants for aortic valve, myocardium, or aorta tissues, the values we selected fall within the ranges reported in studies focusing on aortic walls, the aortic root, or valve leaflets (Holzapfel et al., 2005; Balachandran et al., 2010). Three consecutive cardiac cycles were simulated. Figure 5a shows the pressure changes at the left ventricular exit, in the EOA region, and at the aortic exit. The third cycle is identical to the second, eliminating triggering effects. Figure 5b compares the maximum and relative pressure gradients, showing no qualitative or quantitative differences between the cycles.

### 2.3 Mesh and mesh convergence

The computational mesh for the fluid and solid domains was constructed using COMSOL Multiphysics (Comsol Inc., Stockholm, Sweden). Convergence of the numerical solution was analyzed for the case of normal state. Several finite-element mesh configurations were evaluated during the convergence analysis. The mesh-related information is presented in Table 2. The myocardium is modeled using a standard solid mechanics formulation with linear interpolation of the displacement field, whereas the fluid equations are discretized using a stabilized P_1_+P_1_ finite element pair, that is, continuous piecewise linear shape functions for both the velocity and pressure fields.

**TABLE 2:** Number of mesh elements for convergence analysis.

Left ventricle				
Domains	Mesh 1	Mesh 2	Mesh 3	Mesh 4
Fluid	128 602	240 656	446 638	634 582
Solid	75 960	109 892	147 280	249 442

Aortic valve				
Domains	Mesh 1	Mesh 2	Mesh 3	Mesh 4
Fluid	115 068	235 686	446 638	694 582
Solid	22 684	33 042	47 750	86 098

#### 2.3.1 Left ventricle meshing

**FIGURE 6:**
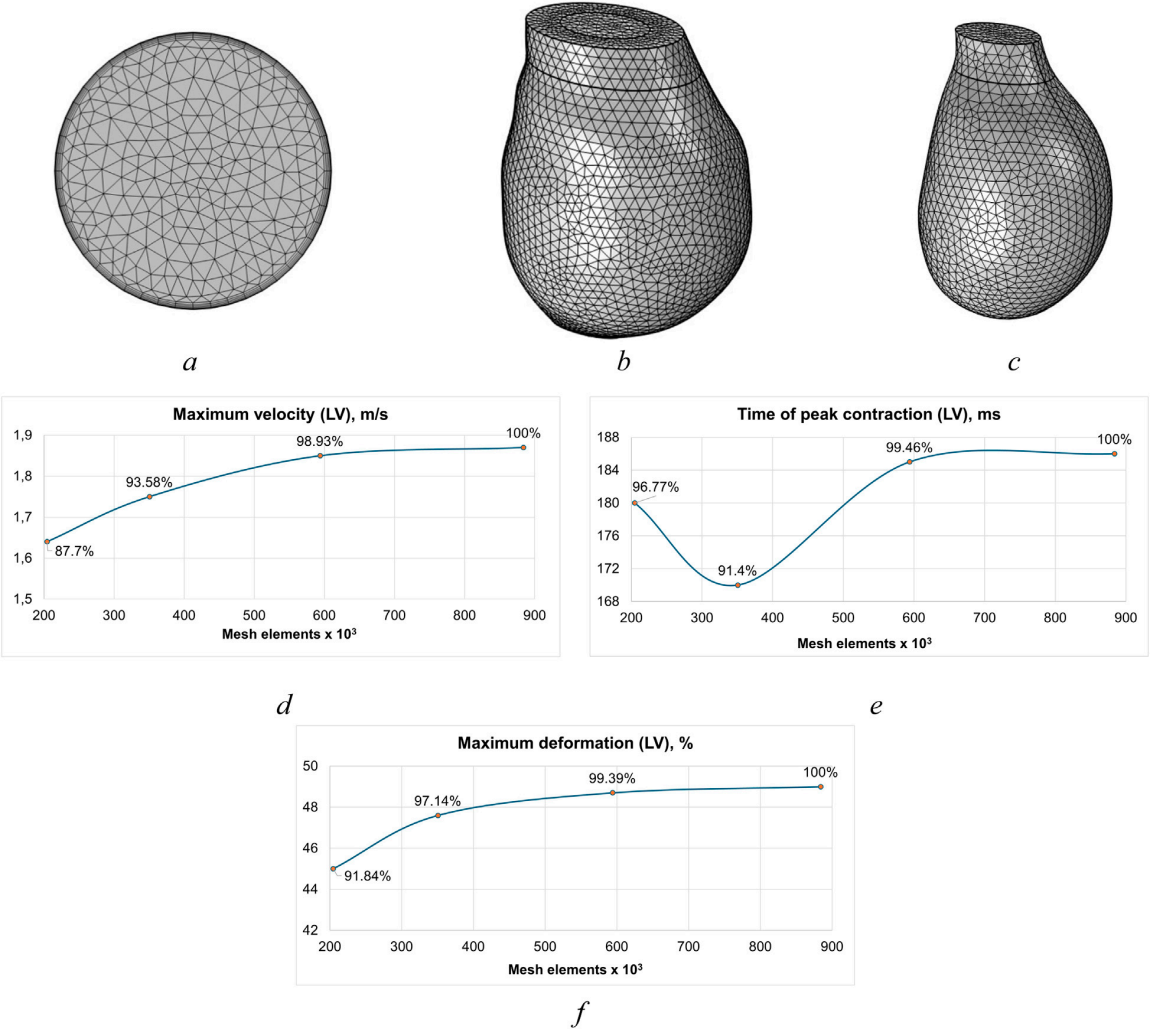
Tetrahedral meshes for fluid and solids with prismatic boundary layers, refinement around leaflets and sinuses, mesh convergence: **(a)** outflow surface mesh **(b)** left ventricle mesh model, **(c)** fluid domain mesh., **(d)** maximum velocity of left ventricle, **(e)** peak left ventricle contraction time, **(f)** maximum deformation of left ventricle.

The minimum element size for both the solid and fluid meshes was determined based on the smallest characteristic dimension of the structure. The convergence analysis allowed for the selection of the optimal element size. Several Mesh independence analyses involved 4 different sizes of mesh. Mesh and mesh convergence plots for the left ventricle domain are presented in Figures 6a–f. Mesh 4 was selected for the computations of left ventricle due to its detail and satisfactory calculation time. The mesh contains 634,582 and 248,442 elements for the fluid and solid domains, respectively. In total, the mesh consists of 883,024 elements, including 849,027 tetrahedral, 15,796 prismatic, 16,124 triangular, 124 quadrilateral, 1,889 edge, and 64 vertex elements. In the fluid domain, five boundary layers with a growth factor of 1.2 are employed. The average element quality is 0.68, and the minimum quality is 0.12.

#### 2.3.2 Aortic valve meshing

**FIGURE 7:**
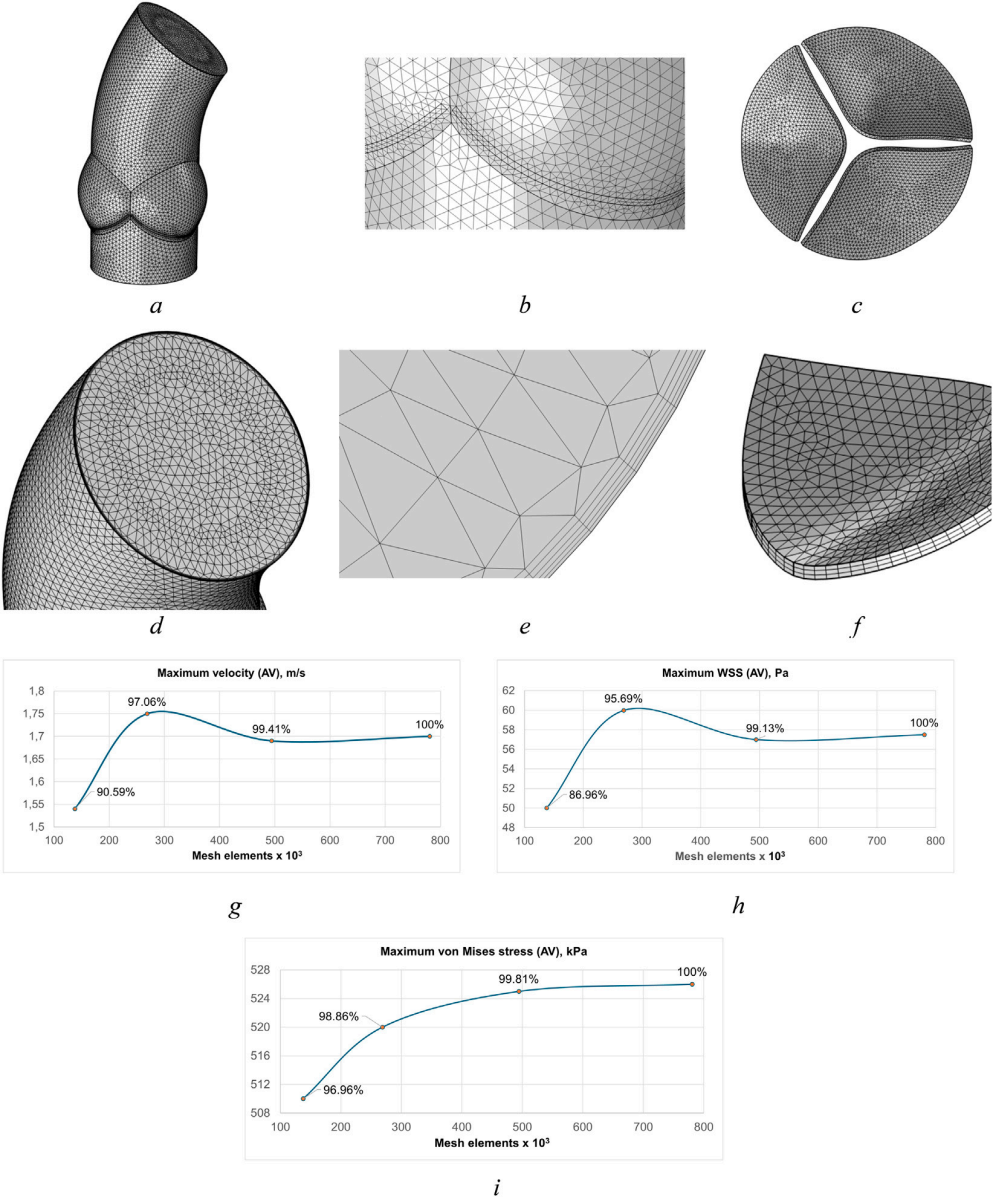
Aortic valve meshes for fluid and solids with prismatic boundary layers, refinement around leaflets and sinuses, mesh convergence: **(a)** aortic root model, **(b)** mesh in aortic leaflets region, **(c)** mesh of leaflets, **(d)** mesh at the outlet, **(e)** inflation layers, **(f)** mesh of leaflet. Mesh convergence analysis: **(g)** maximum velocity of aortic valve, **(h)** maximum wall shear stress of aortic valve, **(i)** maximum von Mises stress of aortic valve.

Aortic valve mesh is shown in Figures 7a–f. The differences in velocity, von Mises stress and wall shear stress results between meshes 3 and 4 was minimal (Figures 7g–i). A mesh convergence study was conducted to ensure that the uncertainty related to spatial discretization was insignificant.

The simulation of the final mesh required more computational time, but the differences in the results were negligible, leading to the conclusion that the results are mesh independent. Consequently, Mesh 3 was selected for subsequent analysis to simulate FSI in aortic valve. Thus, the fluid and solid meshes consisted of 446,638 and 47,750 elements, respectively, and were used for the final calculations. In total, the mesh consists of 494,388 elements, including 475,994 tetrahedral, 8,172 prismatic, 9,443 triangular, 87 quadrilateral, 653 edge, and 39 vertex elements. The optimal fluid mesh was a five-layer mesh with a first-layer thickness of 0.4 mm and a growth rate of 1.2 per layer. The inflation layers provided a more accurate resolution of the boundary layer. Such resolution is essential for some models, such as flows with strong wall effects. The minimum mesh size was 0.2 mm for the solid region and 0.5 mm for fluid flow.

#### 2.3.3 Solver settings

A field-spaced coupling with iterations at each time step is used. The scheme is implicit. The interface ensures that velocities and forces are equal. The fluid and solid are solved alternately until the fields are consistent. At the start of the cycle and during sudden deformations, downward relaxation is enabled. At each time step, subiterations are performed until the discrepancy reaches 1 × 10^−4^ in the L_2_ norm for velocities and pressures and 1 × 10^−4^ for solid body displacements. The maximum number of subiterations is 20. Once the criterion is met, the next time step is taken. The fluid was integrated using an implicit second-order backward differentiation formula (BDF) scheme. The solid body was integrated using an implicit Newmark-type scheme. A common time step was used for both subsystems. The time step was adaptive and varied from 1 × 10^−6^ to 1 × 10^−3^ s. Consistent tangent matrices are specified in the solid body. A stable pressure solution is applied in the fluid. The arbitrary Lagrangian–Eulerian (ALE) mesh moves according to the Laplace equation, with stiffness scaling by the inverse element volume. If necessary, weak numerical damping of interface velocities is enabled. The fluid was solved using unsteady Navier–Stokes equations without a turbulence model, nonlinear Newton–Raphson iteration, and linear solver GMRES with AMG. The solid body was solved using an implicit time scheme.

The LV electromechanics were calculated in COMSOL Multiphysics version 6.2. An anisotropic single-domain model with an implicit time scheme was solved. Newton–Raphson nonlinear iteration was used with a relative error of 1 × 10^−5^ and an absolute error of 1 × 10^−6^. A linear GMRES solver with an algebraic multigrid preconditioner was also used. Solid mechanics were integrated using an implicit Newmark-type scheme. The iteration procedure was constructed as follows: At each time step, the potential propagation was first determined, the myocardial mechanics were calculated, and then the fluid flow was calculated. In the second step, the FSI model of the aortic valve was solved with boundary conditions from the left ventricular ejection. A more detailed scheme for solving the FSI interface is presented in Supplementary Material S2.

## 3 Results

### 3.1 Left ventricle contraction simulation

**FIGURE 8:**
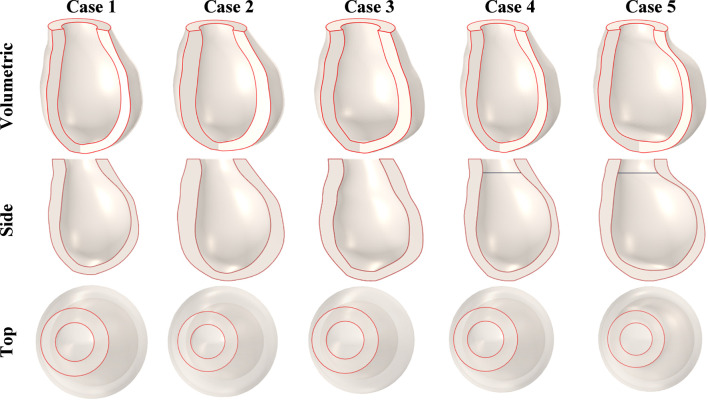
Geometric models of the left ventricle used for the MEFI computational model for cases 1–5.

The models varied in volume from 83.2 mm^3^ to 157.5 mm^3^ and in height from 77.1 mm to 88.1 mm. Additionally, myocardial wall thickness and the shape of the left ventricle were considered as variable parameters. Figure 8 shows the geometric models of the left ventricle for Cases 1–5.

**FIGURE 9:**
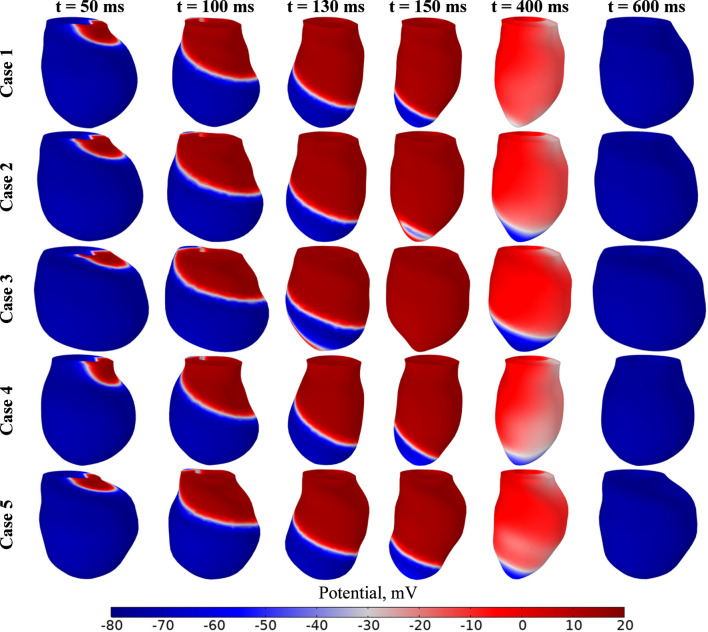
Transmembrane potential maps, evolution from end diastole to valve closure for cases 1–5.

Figure 9 presents the distribution of the electrophysiological potential for each case during the cardiac cycle. We analyzed the dynamics of electrophysiological potential changes at key time steps: 50, 100, 130, and 150 ms for the systolic phase, and 400 and 600 ms for the diastolic phase. At the initial time point, the electrophysiological potential is −80 mV throughout the myocardium except within a rectangular region on the basal surface, where the potential values are −10 mV. During the cardiac cycle, the potential varies between −80 mV and 20 mV, consistent with data reported in the literature (Shimojo et al., 2018; Niederer et al., 2020).

The results indicate a shift in the systolic peak depending on the geometry of the left ventricle. In Case 3, maximum contraction occurs at 150 ms, while in Cases 1, 4, and 5, it occurs at 170 ms, and in Case 2 at 160 ms. Notably, Models 1 and 4 have approximately the same volume but differ in myocardial wall thickness, whereas Model 5 has a significantly larger volume and a thinner wall.

**FIGURE 10:**
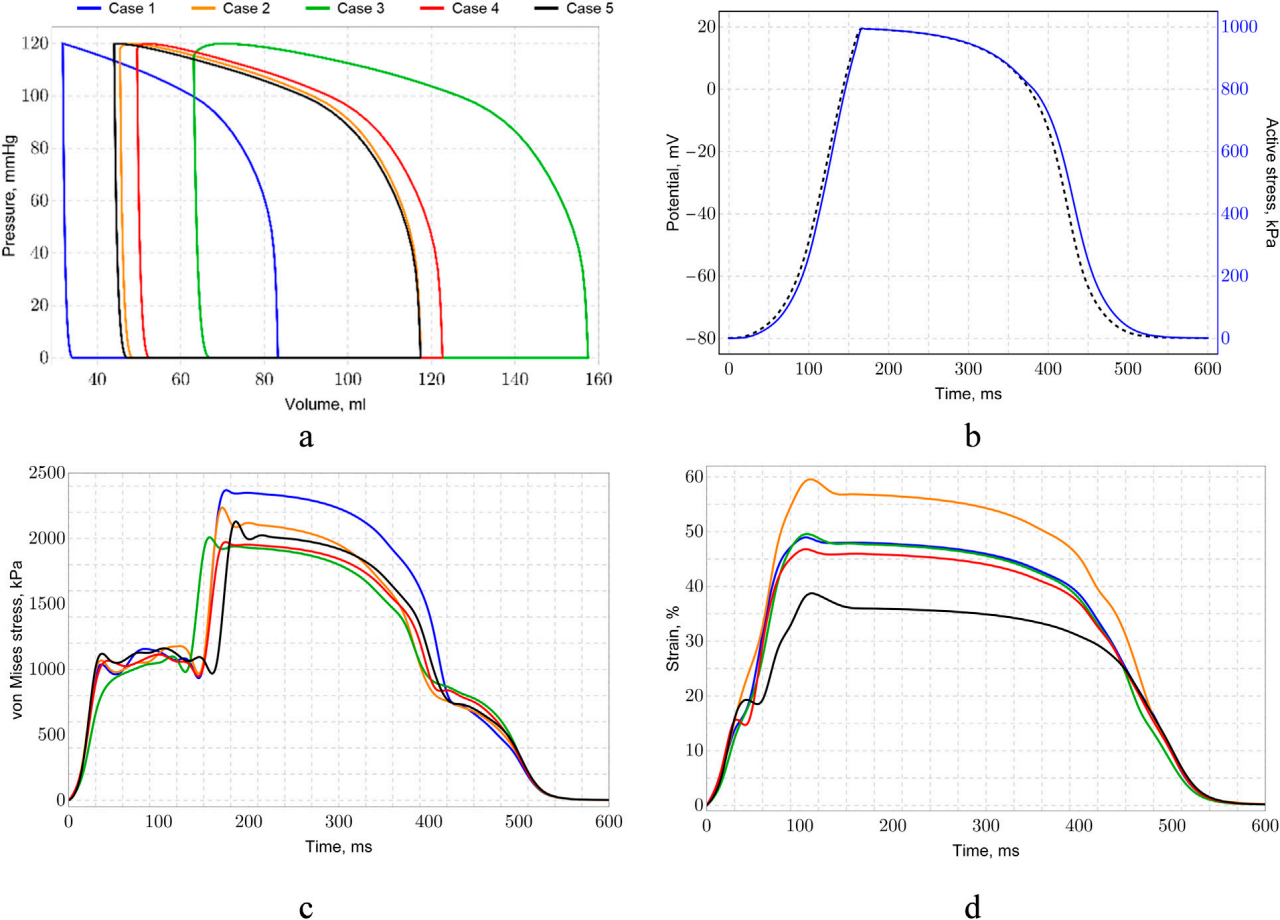
Cardiac cycle pressure–volume (PV) loops and electromechanics: **(a)** PV loops, **(b)** average potential (mV) and active stress (kPa), **(c)** maximum von Mises stress, and **(d)** maximum strain values during the cardiac cycle.

For all designed geometries, the electrophysiological model of the left ventricle was validated using data on volume and ejection fraction, as well as the correlation between volume and pressure changes (Figure 10a). The relationships describing the left ventricle’s contraction–relaxation cycle reveal a link between the integral values of the electrophysiological potential and the active stresses deforming the myocardial walls. Across all cases, variations in electrophysiological potential are minimal. There are no variations in the amplitude of the electrical potential. The peak potential value is the same for all cases. The peak active voltage is also the same. Differences are observed in the activation times and in the thickness and fiber distribution maps. Figure 10b presents the average values of these parameters, showing that active stress lag changes in potential, which is consistent with the excitation-contraction coupling in cardiac muscle (Liu and Paulino, 2017; Humphrey and Schwartz, 2021). In our electrophysiological model, the transmembrane capacity and maximum active voltage values are determined by normalization models and uniform excitation parameters. Therefore, for all cases, peak tuning values exist only during activation and in the form of frontal propagation.

The difference between von Mises stress at time cycle was also presented (Figure 10c). It should be noticed that values for case 1 much differ from another cases at time period between 150 and 500 ms. Moreover, some distinct discrepancies between cases are observed at time period between 25 and 175 ms. At the systolic peak, the maximum deformations of the left ventricle range from 38% to 60% (Figure 10d).

**FIGURE 11:**
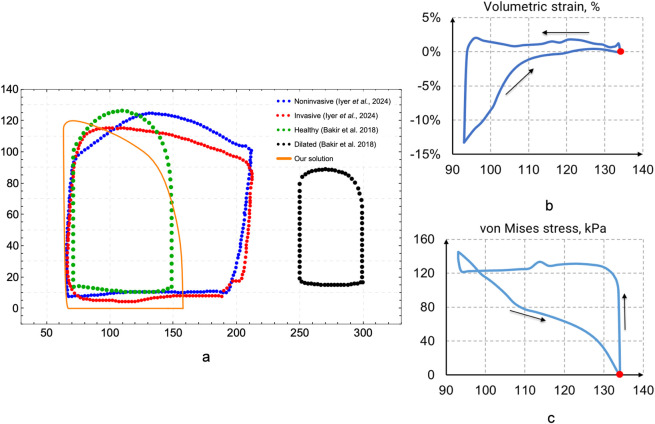
Cardiac cycle pressure–volume (PV) loops comparison of our study with other results **(a)**, volumetric strain **(b)**, von Mises stress for case 1 **(c)**.

Figure 11 a contains cardiac cycle pressure–volume loops for case 1 and comparison with other data. It should be noted that our results are in good correlation with Bakir et al., 2018 results for healthy case. Some discrepancies can be explained by geometry variations. Nevertheless, results show that healthy state can be simulated rather precisely. Moreover, future studies will be devoted to simulation of left ventricle electromechanics at pathology cases such as arrythmia or myocardial infarction. Figures 10b,c exhibit volumetric strain and von Mises stress loops during heart cycle for case 1.

**FIGURE 12:**
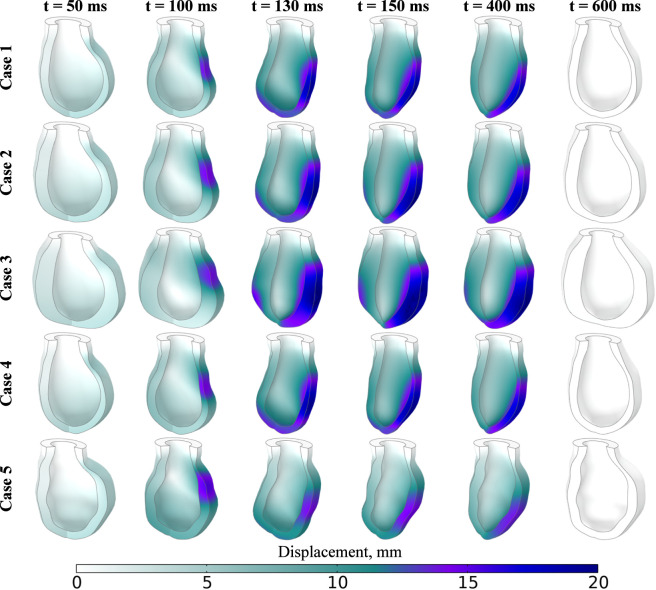
The myocardial walls’ motion during excitation-contraction for Cases 1–5.

The displacement distribution during the contraction–relaxation process is shown in Figure 12, with maximum displacements reaching up to 20 mm, primarily due to the movement of the left ventricular apex at the moment of maximum contraction. These displacement magnitudes align with clinical observations of ventricular motion during systole (Nedadur and Tsang, 2019). This study is also focused on the dynamics of the apex during ventricular contraction.

**FIGURE 13:**
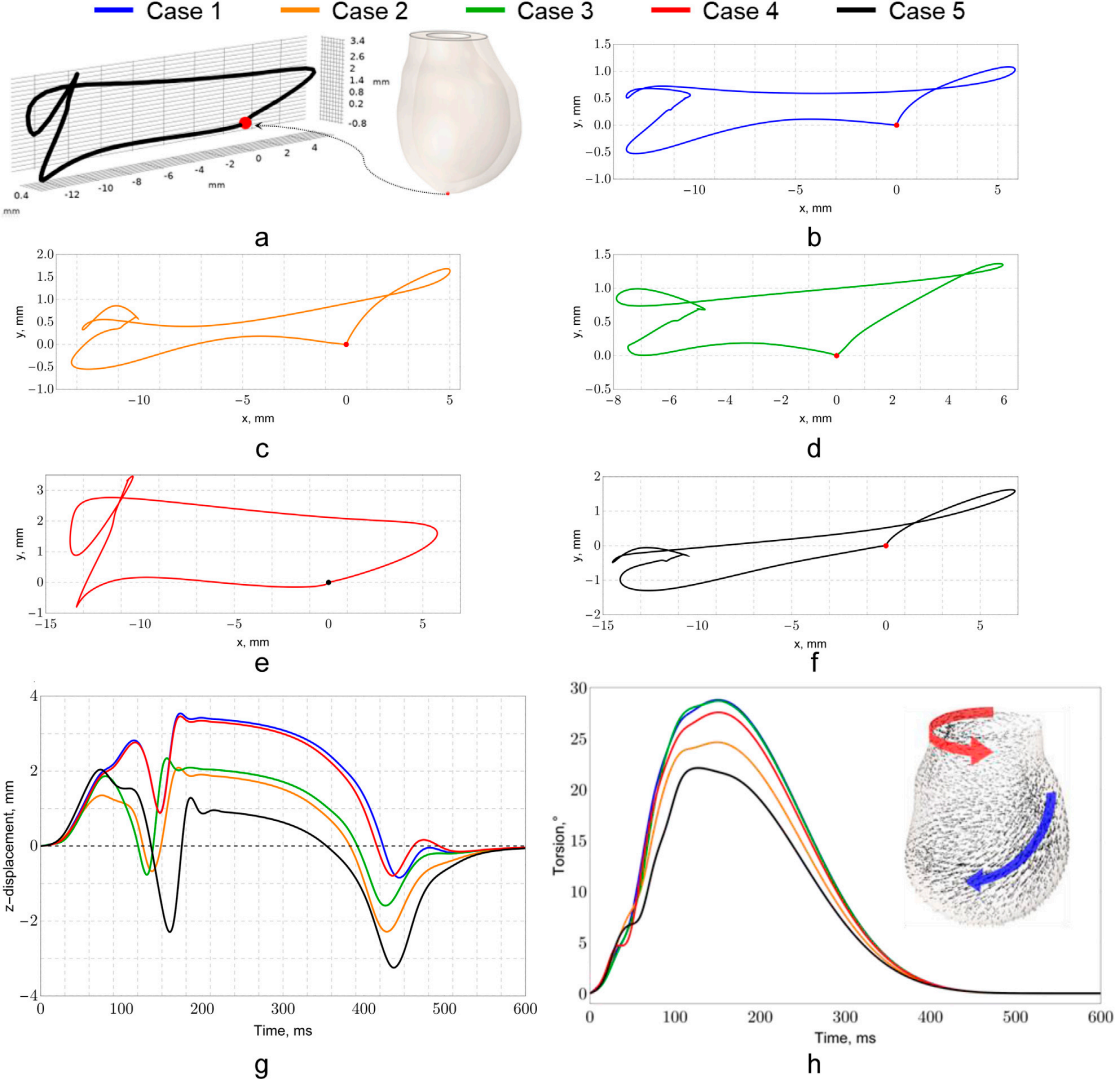
Trajectories of apex motion for Cases 1–5: **(a)** – general scheme, **(b**–**f)** plane trajectories for cases 1–5, **(g)** vertical axis displacement vs. time, **(h)** torsion angle.

Figure 13 illustrates the movement trajectories projected onto the *x*O*y* plane and relative to the vertical axis over time. The displacement of the apex along the *x*-axis does not differ significantly among the cases, ranging from 15 to 20 mm, with the greatest shift occurring in the negative direction. A similar movement pattern is observed along the *y*-axis, though the displacement in this direction is smaller, varying from 2 to 4 mm. The presence of loops in the trajectory indicates the torsion of the myocardium along the fiber direction during contraction, which is consistent with findings in recent studies (Mora et al., 2018).

The torsion angle for each case is shown in Figure 13g. In addition to horizontal movement, it is important to consider the vertical displacement of the apex (Figure 13f), which describes the shortening (compression) of the left ventricle. The greatest shortening is observed in Models 1 and 4, with the apex rising by 3.6 mm, while the smallest occurs in Model 5, where the apex rises by 1.3 mm. Notably, Model 5 also shows the greatest downward displacement of the apex, with a drop of 3.2 mm. The plot illustrates two mechanisms of left ventricular contraction. The first minimum, occurring between 100 and 180 ms, corresponds to the torsion of the left ventricle along the myocardial fibers, resulting in lengthening along the central axis and a downward displacement of the apex. Subsequently, contraction along the central axis occurs, with the apex rising further, leading to a reduction in the internal volume of the ventricle. These observations align with established models of ventricular mechanics and contribute to a deeper understanding of cardiac function (Zores et al., 2019).

**FIGURE 14:**
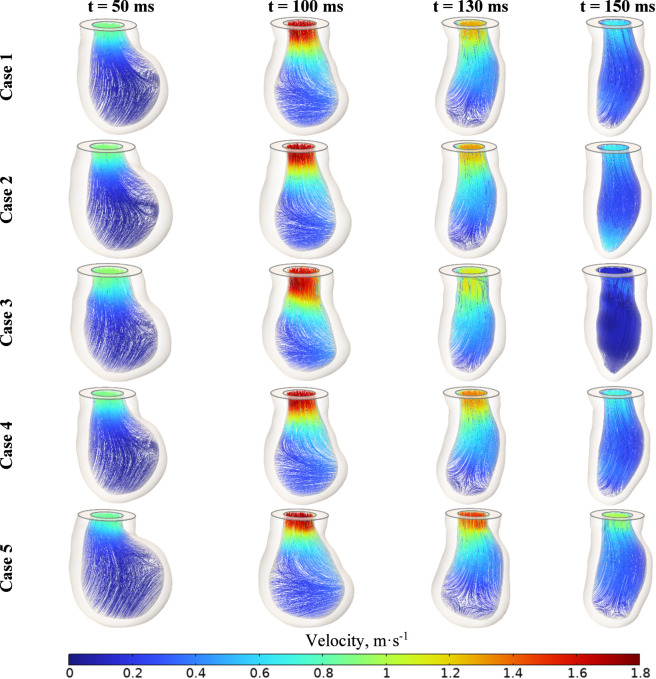
Left ventricular flow velocity streamlines for cases 1–5.

The velocity field was calculated for systolic ejection into the aorta (Figure 14). The valve jet and its shear layers differ in different cases. In Cases 3 and 5, there is stronger twisting of the shear layer and small pockets of recirculation downstream of the leaflets. In Cases 1 and 4, a more uniform jet with limited secondary motion is observed. No significant vortices were detected in the left ventricular tract. Vortex structures occur mainly below the venous constriction and within the sinuses. These differences affect the shear stress on the valve leaflet wall and the stress at the root (Bozinovski, 2019; Lin et al., 2019). The fluid in the LV cavity was solved as a non-stationary, incompressible Newtonian fluid. The setup was implemented in a moving ALE mesh. Boundary conditions on the endocardium were defined by wall velocity from an electromechanical calculation. No slip was set on the walls. Pressure was assumed at the LV outlet. The computation was limited to the systolic interval from the end of diastole to valve closure.

### 3.2 Aortic valve simulation

**FIGURE 15:**
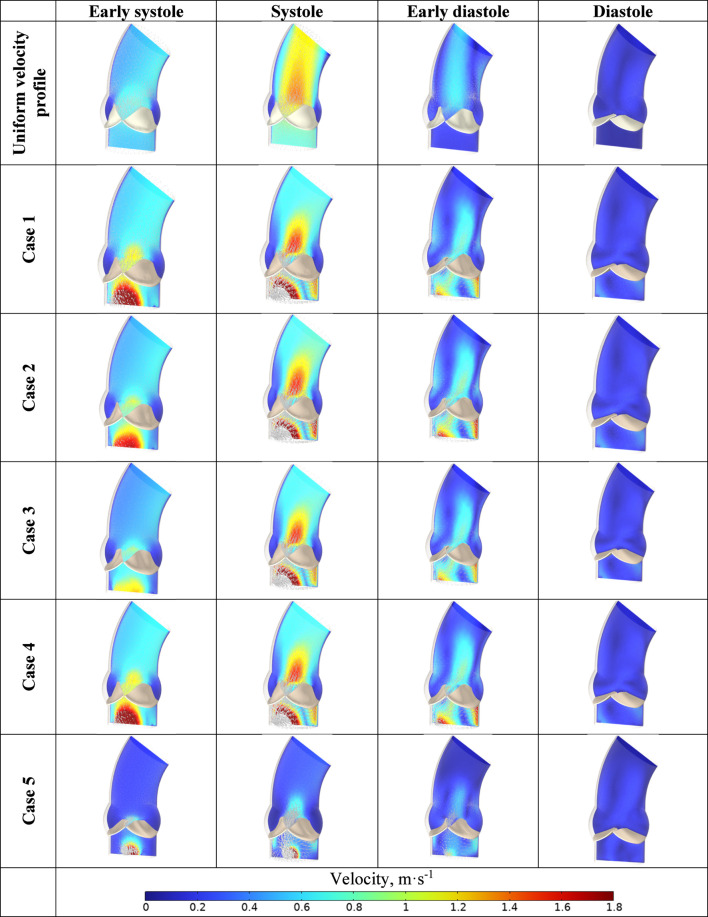
Velocity field distributions in the aorta during the systolic period and early diastole for uniform velocity profile set at inlet and cases 1–5.

The analysis of the resulting flow field distributions plays a key role in this study. Figure 15 shows the flow velocity magnitude and its components for each of the models considered. Additionally, the velocity vector field is depicted at key moments of the cardiac cycle.

The velocity fields obtained at the left ventricular outflow tract were employed as boundary conditions at the inlet of the computational domain for the hemodynamic analysis of the aortic valve. Each simulated case was utilized to examine the impact of the velocity vector field distribution on aortic valve performance.

Flow velocities ranged from 0 to 1.8 m·s^−1^ at the moment of full opening of the aortic valve leaflets, aligning with physiological measurements reported in the literature (Akbari-Shandiz et al., 2019). The obtained distributions demonstrate that the velocity field is unevenly distributed at identical time points during the cardiac cycle. Specifically, Cases 3–5 exhibit lower flow velocities in the early systolic phase compared to Cases 1 and 2. Notably, in Case 5 at 150 ms–corresponding to the onset of diastole–a high velocity is maintained, in contrast to the other cases. The lowest velocities were observed for Case 3 throughout the entire cardiac cycle.

These observations underscore the significant influence of inlet velocity profiles on the hemodynamic environment of the aortic valve, which can affect valve function and potentially contribute to pathologies if abnormal flow patterns persist (Luciani, 2018). Understanding these variations is crucial for the design of patient-specific interventions and prosthetic valves, as well as for predicting disease progression (Hoang-Trong et al., 2021).

An important hemodynamic parameter is WSS. WSS influences the structural and functional integrity of the endothelium and plays a crucial role in processes associated with the development of valvular pathologies, including calcification and stenosis (Gomel et al., 2019; Bańka et al., 2023). In our model, WSS values range from 0 to 20 Pa; however, peak values are confined to localized regions and occur during peak flow velocities at 100 ms.

**FIGURE 16:**
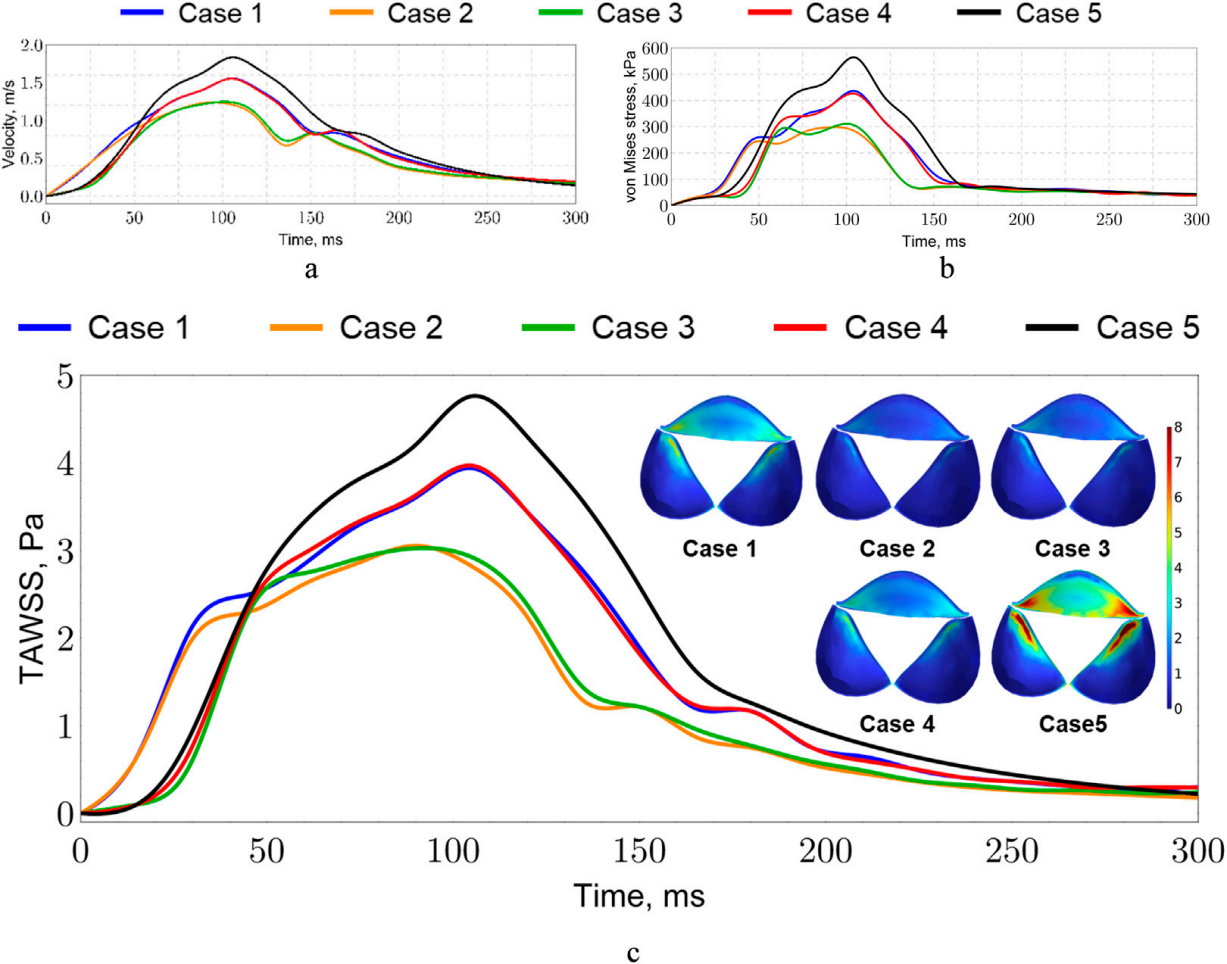
Valve hemodynamics and stress: **(a)** maximum velocity, **(b)** maximum von Mises stress, **(c)** time-averaged wall shear stress.

The maximum von Mises stresses occurring on the aortic valve leaflets did not exceed 600 kPa (Figure 16b), with the highest value observed in Case 5. A clustering of Cases 1 and 4, as well as Cases 2 and 3, is evident in terms of stress levels. A similar correlation is observed in Figure 16a. Cases 1 and 4 share similar geometric characteristics of the left ventricle, which may explain the close values of their parameters. Conversely, Models 2 and 3 have different sizes of the left ventricle and differing inlet flows.

These findings suggest that ventricular geometry significantly influences the stress distribution on the aortic valve leaflets and the flow velocities, consistent with previous computational studies (Hou et al., 2018; Huang et al., 2018). Understanding these relationships is crucial for predicting valve performance and identifying potential areas of high stress that may contribute to valvular pathologies such as calcification and leaflet fatigue (Zebhi et al., 2021; Chioncel et al., 2023).

Similarly, Figure 16c presents the time-averaged wall shear stress (TAWSS). The line graph shows the average over time and surface area, while the distributions demonstrate only time-averaging. The plots in the regions of maximal opening is

The average WSS values for the cases considered lie within the range of 1–3 Pa, which corresponds to normal physiological levels (Hekman et al., 2019). Regions exhibiting abnormal WSS values are more susceptible to calcification formation, consistent with findings reported in recent studies (Sun et al., 2012).

Cases 2 and 3 represent normal physiological conditions, as the TAWSS values throughout the cardiac cycle do not exceed 3 Pa, and no regions with high stress values are observed. In contrast, Cases 1, 4, and 5 tend to simulate pathological scenarios, as the TAWSS values are in the range of 4–5 Pa, and regions of stress localization are observed on the valve leaflets, where calcification may subsequently develop. This observation aligns with studies indicating that elevated TAWSS can contribute to valvular calcification and the progression of aortic stenosis (Kamath and Pai, 2011; Gomel et al., 2019).

These findings highlight the importance of hemodynamic factors in valvular health and disease. Elevated shear stress may lead to endothelial dysfunction and promote the initiation of calcific nodules on the valve leaflets, underscoring the need for early detection and potential therapeutic interventions (Bańka et al., 2023).

### 3.3 Validation study

The electrophysiological model encompasses a considerable number of parameters (Table 1). Some of these possess a clear physical interpretation (for instance, reflecting myocardial contractility or excitation conduction properties), while others serve as model coefficients introduced to accurately replicate complex physiological processes. Determining appropriate parameter ranges and establishing systematic approaches for their calibration are among the goals of our work.

In the initial phase of validating the LV model, we focused on global indicators such as the ejection fraction (EF) and pressure–volume (PV) loops. However, these integral metrics alone do not fully characterize the local dynamics of myocardial contraction and relaxation. We therefore further examined how varying certain parameters affects LV torsion—a key contributor to cardiac pumping efficiency. Among the entire parameter set, those describing external loading and different components of wall deformation are of particular importance: $\eta_{1}$ controls radial inward movement, $\eta_{2}$ governs longitudinal shortening, and $\eta_{3}$ primarily influences the twisting motion.

**FIGURE 17:**
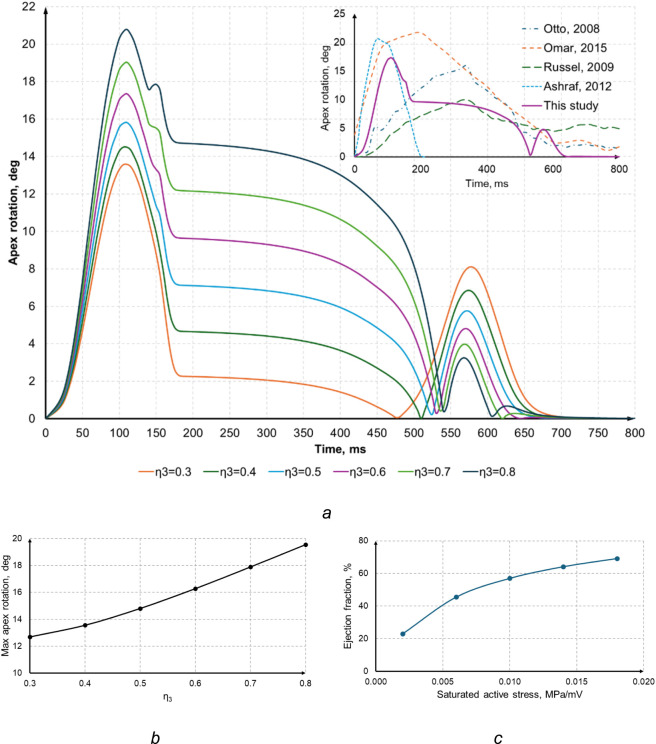
**(a)** Apex rotation angle at different $\eta_{3}$ values and comparison with literature data at $\eta_{3}=0.6$ ; **(b)** maximum apex rotation depending on $\eta_{3}$ ; **(c)** ejection fraction at different values of saturated active stress (*k*).

Figures 17a,b illustrates how the apex torsion changes when $\eta_{3}$ varies from 0.3 to 0.8, with the maximum twist angle ranging between 13.5° and 21°. The figure also compares the apex rotation against published data at a fixed parameter value (e.g., 0.6), showing that our model successfully reproduces the essential torsional mechanics of the LV and aligns well with physiological estimates.

Matching the computed results to the observed EF can, in particular, be achieved by proportionally adjusting the parameters $\eta_{1},\eta_{2},\eta_{3}$ or by tuning the saturated active stress coefficient ( k ). Bakir et al., 2018 computed the ejection fraction evolution and defined velocity streamlines and distribution of electrophysiological potential at $\eta_{1}=0.1$ , $\eta_{2}=0.2$ , and $\eta_{3}=0.6$ . EF was found to rise from 23% to 70%, aligning with clinical measurements typically associated with either healthy or weakened myocardium. In addition to parametric studies, the LV model was validated using numerical and clinical data. The streamlines and transmembrane potential from Bakir et al. (2018) are in qualitative agreement with the patterns in Figure 11. The potential range in our Figure 7 is from −80 to 20 mV, which is consistent with the obtained values. Spatial differences from the reference values arise in studies including radially distributed Purkinje fibers. Activation does not depend solely on fiber alignment. We solve an anisotropic monodomain model with a conductivity tensor aligned with the direction of the fibrous layer and normal to the layer. This results in faster propagation along the fibers and slower propagation across the fibers and through the wall. The Purkinje network is not included. Endocardial activation is applied as a brief stimulus to selected areas of the endocardium with early foci on the septum and apical side. This choice reflects the scope of the systolic driver for inflow to the valve, rather than a complete electrophysiological study. The PV curves in Figure 9a agree with the literature data both qualitatively and quantitatively (Iyer et al., 2024), which combines invasive and noninvasive pressure and volume assessments. The left ventricular wall displacements in Figure 9 are consistent with the data of Obermeier et al. (2022). In particular, the apex movements are in the range of 4–6 mm, which is also confirmed by our model. The fiber orientation field was verified using *in vivo* data published by Li et al. (2025). Moreover, presented approach can take into account any fiber orientation, which can be derived from imaging data.

## 4 Discussion

In this study, we have developed an advanced hemodynamic model of the aortic valve integrated with an electrophysiological model of the left ventricle. This integration enables the establishment of realistic boundary flow conditions at the inlet of the computational domain, thereby enhancing the fidelity of our simulations. The left ventricle model provides detailed results for the distribution of electrophysiological potentials, wall movements, and velocity fields within the myocardium. Additionally, we computed integral characteristics such as stresses, strains, and flow velocities to gain comprehensive insights into cardiac mechanics. The calculations were performed for five geometric model cases, each representing various shapes and sizes of the left ventricle, to assess the impact of anatomical variations on cardiac function.

### 4.1 Left ventricle contraction simulation

The parameters of these models were identified based on the correlation between pressure–volume relationships and ejection fraction, as reported in the literature (Baillargeon et al., 2014; Chabiniok et al., 2016; Janssens and Bovendeerd, 2024). The myocardial wall motion in our model is illustrated in Figure 10, while the trajectories of the left ventricular apex movement are further analyzed in Figure 10. Notably, the ranges of motion qualitatively and quantitatively align with the data presented by Obermeier et al. (2022), who investigated left ventricular function using CT data from patients followed by numerical processing. The average movements observed in our study did not exceed 10 mm, with the largest deviation recorded at the apex, measuring 17 mm. Our results indicate that maximum displacements do not exceed 20 mm, primarily occurring at the apex, as shown in Figure 11, and along the lateral wall of the left ventricle. This finding is consistent with results from studies analyzing MRI data (Ibrahim, 2011; Smiseth et al., 2016), reinforcing the validity of our model.

Following the calculation of the electrophysiological model and the assessment of stresses associated with the contraction and relaxation of the left ventricle, we proceeded to calculate the flow velocity fields (Figure 12). Maximum velocities were observed at the outlet of the left ventricle, which were further analyzed in terms of absolute values and the contributions of the velocity vector components, including the direction of the vector field. The simulation results indicate that flow velocities within the left ventricle vary from 0 to 1.8 m·s^−1^, surpassing the values reported in some previous studies where echocardiographic and Doppler measurements indicated velocities of approximately 0.8 ± 0.21 m·s^−1^. Conversely, other studies using Doppler ultrasound and transesophageal echocardiography have recorded peak velocities ranging from 1.4 to 2.2 m·s^−1^ (Vahanian et al., 2022). The higher velocities observed in our simulations may be attributed to the simplified assumptions in the model or variations in ventricular geometry and contractility.

It is important to note that increased flow velocities may correlate with the development of various pathologies, particularly aortic stenosis. This suggests that our model can capture hemodynamic conditions that are relevant to pathological states. The ability to simulate such conditions is crucial for understanding the progression of cardiovascular diseases and for designing effective therapeutic interventions.

### 4.2 Aortic valve simulation

Similarly, the solutions obtained in the previous stage were integrated into the mathematical model of the aortic valve as boundary conditions for the flow.

The velocity fields obtained, as presented in Figure 13, qualitatively and quantitatively agree with literature data. The maximum flow velocities in the aortic valve for the considered cases range from 1.2 to 1.8 m·s^−1^, indicating the model’s capability to simulate various scenarios of normal and pathological conditions. According to the literature, in aortic stenosis, velocities can reach 3–4 m·s^−1^ (Baumgartner et al., 2017). In the current setup, the condition of the valve leaflets was considered healthy, and corresponding parameter values were used to define the material model. Stenotic leaflets become stiffer, narrowing the lumen and increasing flow velocities (Garcia et al., 2019). Our model can account for these conditions by modifying the properties of the material model describing the biomechanics of the leaflets, as we have previously demonstrated in our work (Pil et al., 2023).

An open question remains regarding the turbulent nature of the flow in the aorta. It is more precise to refer to the presence of vortex structures that arise in the ascending aorta (Pangelina et al., 2025), which can occur even under the laminar assumption employed in our model. The characteristic Reynolds numbers do not exceed 4,000, indicating a transitional rather than a fully turbulent regime (Cheng et al., 2025). Turbulence effects are especially important when modeling pathological conditions such as aortic stenosis (Stein and Sabbah, 1976; Manchester et al., 2021). Figure 13 shows the formation of recirculating flows in the diastolic phase.

Our calculation is primarily aimed at resolving large-scale flow features (including spiral or secondary flows), whereas capturing finer turbulent structures would require substantially higher mesh resolution and more advanced turbulence models (URANS, LES, or others).

The calculated WSS also showed agreement with literature data. WSS is an important hemodynamic parameter in describing blood flow processes in the heart and arteries. These stresses affect the structural and functional integrity of the endothelium, as well as processes associated with the development of valve pathologies, including calcification and stenosis (Kazik et al., 2021). Normal WSS promotes the release of vasoactive substances by endothelial cells, such as nitric oxide, which regulates vascular tone and prevents thrombosis (Katoh, 2023). Optimal WSS maintains the healthy morphology and mechanical properties of the valve, preventing degenerative changes. Alterations in WSS can lead to activation of inflammatory pathways, stimulating cell proliferation and calcium deposition, which leads to valve pathologies (Zhou et al., 2023).

Typically, WSS values vary from 1 to 3 Pa, values exceeding 4 Pa can lead to mechanical damage to the endothelium and initiate calcification processes (Xu et al., 2023). In our model, both scenarios are observed: Cases 2 and 3 describe the normal situation where WSS does not exceed 3 Pa, while Cases 1, 4, and 5 exhibit higher stress values and localized regions on the valve leaflets. These high-stress regions may correspond to areas susceptible to calcification and the onset of aortic valve disease.

The calculations show a consistent asymmetry between the ventricular and aortic sides of the valves. On the ventricular side, the average and peak shear are higher, with maxima along the attachment line and in the central area of the belly. On the aortic side, elevated values are more common at the free edge and in the segments between the free edge and the commissures. Curvature and unsteadiness increase the variability of flow at the valve surface. Increased free-edge bending and flutter decrease local TAWSS, primarily in the ventricle and near the commissures. The peaks of the indicators coincide with areas of high mechanical stress, indicating a risk of tissue degradation and thrombogenic susceptibility. The WSS map and derived indicators are key descriptors of vulnerability, explaining why small changes in the inflow profile or geometry can shift the location of dangerous areas on both valve surfaces (Tsolaki et al., 2023; Costa et al., 2025).

### 4.3 Model coupling

Our model’s key feature is the integration of left ventricular electrophysiology calculations, from which stresses are determined and transmitted to the contraction–relaxation problem. The results of this step yield velocity fields at the outlet of the left ventricle, which are then used as boundary conditions for the hemodynamic analysis of the aortic valve.

This comprehensive approach to modeling physiological processes allows us to account for many more factors than existing models typically consider. Similar ideas are found in the literature, where authors use 4D MRI to set boundary conditions, or they perform modeling within a CFD framework without considering fluid–structure interactions (Mittal et al., 2016). By incorporating an electrophysiological model into the mechanical work of the left ventricle and aortic valve, we can simulate various patient conditions by altering the initial data and model parameters.

In cases of ischemic heart disease, insufficient blood supply to the heart muscle leads to hypoxia of myocardial cells, impairing their ability to conduct electrical impulses (Heusch, 2016). Our model is capable of computing this field based on the LV geometry and the mechanical and electrophysiological properties of myocardial tissue. Nonetheless, the model’s structure, incorporating electrophysiology, active contraction, and an FSI approach, allows for the future exploration of various physiological and pathophysiological parameters, such as reduced contractility in cardiomyopathies (Davey et al., 2024).

The model is capable of simulating hypertrophy and dilation by increasing wall thickness (concentric hypertrophy), increasing internal volume (dilation), or a combination of both (Garg et al., 2017). This approach has already been used in several studies evaluating the effects of pathologic changes on cardiac mechanics and hemodynamics (Aimo et al., 2024; Jumadilova et al., 2024). In our system, we can modify the initial LV geometry to reproduce mild or severe dilation as well as different forms of hypertrophy.

Thanks to the developed algorithm for constructing parameterized geometries of the left ventricle, we can simulate its operation under conditions of hypertrophy and dilation, in which the wall thickness and cavity size change (Kohles et al., 2009; Kheyfets et al., 2015). This flexibility allows for the study of pathological conditions such as left ventricular hypertrophy, where increased wall thickness affects cardiac function, or dilated cardiomyopathy, characterized by enlargement of the ventricular cavity and reduced contractility.

Our model’s ability to adjust geometric and electrophysiological parameters provides a powerful tool for personalized medicine, enabling the simulation of patient-specific scenarios and the exploration of therapeutic strategies. Future work may involve coupling this model with clinical data to validate its predictive capabilities and extend its application to other cardiac pathologies.

### 4.4 Study limitations

Despite the extensive functionality and potential for further development of the proposed model, it has several limitations that we plan to address in future work. Since the problem is formulated within the context of fluid-solid interaction, we must maintain the continuity of the medium, so finite elements of the fluid region always remain between the leaflets. However, contact interaction between the leaflets is additionally specified at a distance equal to the finite element, transmitting stress from one leaflets to the other. Firstly, the proposed electrophysiological model, based on the works of Nash and Panfilov with modifications by Bakir et al. (2018) and our revisions, contains a large number of parameters that require identification. We validated the model based on data concerning pressure and volume changes, controlling the ejection fraction, which does not account for changes in field variables. A promising direction here is the comparison with ultrasound data using speckle tracking techniques, as presented in studies by Mor-Avi et al. (2011) and Voigt et al. (2015), as well as incorporating 4D MRI studies.

Secondly, an important effect during left ventricular contraction is its torsion along myocardial fibers, which increases the speed and volume of ejection and induces a helical flow pattern. Helical (spiral) blood flow reduces resistance within the vessels, resulting from a more uniform velocity distribution across the vessel cross-section, which decreases energy losses during blood flow (Lo et al., 2007). The helical motion helps maintain blood movement with lower energy expenditure, which is crucial for the efficient functioning of the cardiovascular system.

Helical flow contributes to sustaining laminar blood flow, preventing turbulence formation. Turbulence can lead to endothelial damage and promote thrombosis (Stein and Sabbah, 1974). The twisted flow ensures a more uniform distribution of shear stresses on the endothelium, stimulating normal vascular wall function and maintaining vascular health. Proper mechanical stimuli from the helical flow promote the release of vasoactive substances by the endothelium, such as nitric oxide, which regulates vascular tone (Cheng et al., 2006).

Furthermore, helical flow reduces mechanical load on the aortic valve leaflets, potentially prolonging their effective function and reducing the risk of valvular diseases (Wong et al., 2025). Correct flow dynamics facilitate complete valve closure after systole, preventing regurgitation into the left ventricle. Given that the aorta has a curved shape and branches into smaller arteries, helical flow passes through these structures more efficiently, minimizing turbulence and optimizing blood flow (Kilner et al., 2000). The twisted flow contributes to the formation of stable vortex structures, which can play a role in distributing blood to the branches of the aorta. Efficient and stable blood flow ensures a more uniform supply of organs and tissues with oxygen and nutrients. Laminar helical flow reduces the likelihood of platelet aggregation and thrombus formation.

This study exclusively uses LV electromechanics to describe the systolic phase to generate a time-resolved physiological flow velocity field for the subsequent aortic valve FSI. The computational window spans the period from end diastole (ED) to aortic valve closure; overt mitral inflow and diastolic filling are not modeled. Geometrically, the simulation starts with the mapped ED configuration, which we consider as an effectively prestressed initial state; before ejection, we perform a short-term quasi-static equilibration at ED pressure with zero active stress to ensure mechanical consistency in the ED. The model then goes through isovolumic contraction and ejection until aortic closure. Indeed, the ED is not a truly unloaded state, so we limit the interpretation of the LV results to the systolic interval. Sensitivity testing replacing ED-based initialization with a zero-inflow early relaxation window revealed no significant changes in the valve system metrics presented here: jet time, velocity magnitude, WSS, TAWSS, and von Mises stress. Full restoration of the unloaded state by pre-stressing is planned for future studies of LV mechanics beyond valve actuator functions. Diastolic filling is not modeled. Modeling begins with the end-diastolic configuration and covers isovolumic contraction and ejection until aortic valve closure. The dynamics of mitral inflow and filling are beyond the scope of this study. Future work will include explicit diastolic filling of the mitral inflow tract and bidirectional coupling between the left ventricle and aortic valve to study behavior over a full cycle.

PV trajectories are similar across cases. Pressure is set by a common outflow model and a single electrophysiologic driver. As a result, differences in pressure between hypertrophy and dilation are not fully reflected. Conclusions for valve metrics apply to fixed loading. Assessing afterload effects requires a separate series with modified outflow parameters and calibration to clinical pressures.

Finally, the influence of the velocity vector field distribution on the hemodynamics of the aortic valve remains an open question. To further enhance the aortic valve model, it is necessary to transition to patient-specific geometries, incorporating fluid–structure interactions not only on the valve leaflets but also on the walls of the aorta. This advancement would allow for more accurate simulations of physiological conditions and improve the model’s predictive capability in clinical applications.

### 4.5 Concluding remarks

In this study, we developed a hemodynamic model of the aortic valve that integrates left ventricular electrophysiology with fluid–structure interactions. The model combines electrical activation and conduction with a hyperelastic, anisotropic myocardium and accounts for active contraction and passive wall motion. Parameterized LV geometry reproduces a wide range of sizes and shapes, including hypertrophy and dilation (Basso et al., 2021; Hermida et al., 2023). LV modeling provides time-resolved inlet boundary conditions for the FSI valve model. While the steps are sequential rather than fully coupled, this is the first systematic demonstration of how LV shape and state influence valve biomechanics. Previous studies typically considered the valve with a simplified inflow or ventricle without valve deformation or electrophysiology. This combined representation opens a new perspective on LV–valve interactions.

This approach reproduces velocity fields and stress distributions consistent with literature and clinical data. Realistic boundary conditions based on LV mechanics improve the accuracy of valve hemodynamics. Accounting for LV torsion helps reconstruct helical flow patterns, which reduce energy loss. The results demonstrate how LV anatomy and pathology affect blood flow and leaflet loading.

Visualization-based models assist in selecting the type of prosthesis and procedure, as well as in planning additional reconstructions. The integration of myocardial electrophysiology allows changes in conduction and excitability to represent bundle branch block, ischemia, and fibrosis. These disturbances alter wall motion and local blood flow and can exacerbate valve dysfunction. The model allows for *in silico* assessment of their impact on valve function and leaflet loading (David and Ivanov, 2003; Kwiecinski et al., 2021).

Further research will improve patient specificity using 4D MRI and speckle-tracking echocardiography. Moreover, it will expand FSI to include aortic wall dynamics to assess systemic hemodynamic effects, enhance prognostic value, and improve clinical applicability to valves. These models allow for the assessment of how changes in LV geometry, prosthetic valve implantation, or fibrotic remodeling affect overall cardiac function without the risk of intervention, which is valuable in cases of multiple malformations, congenital anomalies, and postoperative conditions (Sun et al., 2014; Fumagalli et al., 2024).

## Data Availability

The raw data supporting the conclusions of this article will be made available by the authors, without undue reservation.
