# Supplementary material for: Mechano-electrical-fluid interaction left-ventricle model for numerical evaluation of aortic valve hemodynamics
Source: Front Bioeng Biotechnol. 2026 Jan 7;13:1713023. doi: 10.3389/fbioe.2025.1713023 (PMC12819714; doi:10.3389/fbioe.2025.1713023)
Supplement: Supplementary file 1 [file Supplementaryfile1.docx]

**Parameters for electrophysiological left ventricular model**

| **Parameter** | **Value** | **Description** |
| --- | --- | --- |
| $\chi_{m}$ | 1 | Area to volume ratio, m^-1^ |
| $C_{m}$ | 1 | Transmembrane capacitance per unit area, F·m^-1^ |
| $d_{ani}$ | 0,0001 | Anisotropic conduction velocity, mm^2^·s^-1^ |
| $d_{iso}$ | 0,001 | Isotropic conduction velocity, mm^2^·s^-1^ |
|  | 100 | Conversion factors, mV |
|  | -80 | Conversion factors, mV |
|  | 0,6 | Resting potential of stretched-activated channels |
| $k$ | 0,01 | Saturated active stress, Pa·mV^-1^ |
| $\tau_{0}$ | 1 | Time parameter, ms |
| $t_{0}$ | 0 | Time parameter, ms |
| $t_{1}$ | 400 | Time parameter, ms |
| $\eta_{1}$ | 0.4 | Active stress scaling |
| $\eta_{2}$ | 0.4 | Active stress scaling |
| $\eta_{3}$ | 0.4 | Active stress scaling |
| $\alpha$ | 0.01 | Aliev-Panfilov parameter |
| $b$ | 0.15 | Aliev-Panfilov parameter |
| $c$ | 8 | Aliev-Panfilov parameter |
| $\gamma$ | 0.002 | Aliev-Panfilov parameter |
| $\mu_{1}$ | 0.2 | Aliev-Panfilov parameter |
| $\mu_{2}$ | 0.3 | Aliev-Panfilov parameter |
| $\varepsilon_{0}$ | 100 | Rate constants of contraction, s^-1^ |
| $\varepsilon_{1}$ | 1000 | Rate constants of contraction, s^-1^ |
| $Ф_{r}$ | -80 | Resting potential, mV |
| $\zeta$ | 1 | Transition rate, mV^-1^ |
|  | 0 | Phase shift, mV |

**Mechanical model parameters of left ventricle**

| **Parameter** | **Value** | **Description** |
| --- | --- | --- |
| $\mu$ | 200000 | Lamé parameter, Pa |
| $\lambda$ | 500000 | Lamé parameter, Pa |
| $\rho$ | 1370 | Myocardial density, kg·m^-3^ |
| *k_1_* | 1685 | Fiber direction material property, Pa |
| *k_2_* | 15.779 | Fiber direction material property |
| $\tau_{epi}$ | -1.0472 | Epicardial fiber angle at basal plane, rad |
| $\tau_{endo}$ | 1.0472 | Epicardial fiber angle at basal plane, rad |

**Mechanical model parameters of aortic valve**

| **Parameter** | **Value** | **Description** |
| --- | --- | --- |
| $\rho$ | 1100 | Leaflet density, kg·m^-3^ |
| C_10_ | 30.03 | Material model parameter, kPa |
| C_01_ | 3.47 | Material model parameter |
| *k_1_* | 74.5 | Fiber direction material property, Pa |
| *k_2_* | 63.19 | Fiber direction material property |
| *κ* | 0.2 | Dispersion level parameter |
