# Supplementary material for: Mechano-electrical-fluid interaction left-ventricle model for numerical evaluation of aortic valve hemodynamics
Source: Front Bioeng Biotechnol. 2026 Jan 7;13:1713023. doi: 10.3389/fbioe.2025.1713023 (PMC12819714; doi:10.3389/fbioe.2025.1713023)
Supplement: Supplementary file 2 [file Supplementaryfile2.docx]

**Supplementary material 2**

**Numerical procedure**

To numerically solve the fluid-structure interaction (FSI) problem using the finite element method (FEM), the governing equations must first be transformed into their weak forms and then discretized appropriately. This transformation is essential to apply the FEM, which relies on the weak formulation of differential equations (Averweg *et al.*, 2024).

To derive the weak form of the governing equations, we multiply each equation by suitable test functions and integrate over the respective domains (Zimbrod, Fleck and Schilp, 2024). Specifically, we select test functions $\mathbf{v}_{\mathbf{f}}$ for the fluid velocity and $q$ for the fluid pressure, belonging to appropriate function spaces that satisfy the necessary boundary conditions and ensure numerical stability.

Multiplying the Navier–Stokes equations by these test functions and integrating over the fluid domain $\Omega_{f}$ yields the weak form of the fluid dynamics equations, all the body force is neglected:

| $\int_{\Omega_{f}} \rho_{f}\left( \frac{\partial\boldsymbol{\upsilon}_{\boldsymbol{f}}}{\partial t}\cdot\mathbf{v}_{\boldsymbol{f}}+\left[ \boldsymbol{v}_{f}-\boldsymbol{u}_{f} \right]\cdot\nabla\boldsymbol{v}_{f}\cdot\mathbf{v}_{\boldsymbol{f}} \right)d\Omega+\int_{\Omega_{f}} \mu_{f}\nabla\boldsymbol{\upsilon}_{\boldsymbol{f}}:\nabla\mathbf{v}_{\boldsymbol{f}}d\Omega-\int_{\Omega_{f}} p\nabla\cdot\mathbf{v}_{\boldsymbol{f}}d\Omega+\int_{Г} \mathbf{v}_{\boldsymbol{f}} \cdot\left( \boldsymbol{\sigma}_{f}\cdot\mathbf{n} \right)dГ=0.$ | (1) |
| --- | --- |

The incompressibility condition is enforced weakly:

| $\int_{\Omega_{f}} q\nabla\cdot\mathbf{\upsilon}_{\mathbf{f}}d\Omega=0.$ | (2) |
| --- | --- |

Similarly, selecting a test function $\mathbf{v}_{\mathbf{s}}$ for the solid displacement and integrating over the solid domain $\Omega_{s}$, we obtain:

| $\int_{\Omega_{s}} \rho_{s}\frac{\partial^{2}\boldsymbol{u}_{\boldsymbol{s}}}{\partial t^{2}}\cdot\mathbf{v}_{\boldsymbol{s}}d\Omega+\int_{\Omega_{s}} \boldsymbol{\sigma}_{\boldsymbol{s}}:\nabla\mathbf{v}_{\boldsymbol{s}}d\Omega=\int_{Г} \mathbf{v}_{\boldsymbol{s}} \cdot\left( \boldsymbol{\sigma}_{s}\cdot\mathbf{n} \right)dГ.$ | (3) |
| --- | --- |

The coupling conditions at the interface $\partial\Omega_{fsi}$ are incorporated into the variational formulation, either through interface integrals or by appropriately choosing the test functions. The fluid velocity, pressure and solid displacement with its test functions approximates using shape functions:

| $\boldsymbol{\upsilon}_{\boldsymbol{f}}\left( \boldsymbol{x},t \right)\approx\sum_{i=1}^{N_{v}} \boldsymbol{U}_{i}\left( t \right)N_{i}\left( \boldsymbol{x} \right),$ | (4) |
| --- | --- |
| $p\left(\mathbf{x},t\right)\approx\sum_{j=1}^{N_{p}} P_{j}\left(t\right)M_{j}\left(\mathbf{x}\right),$ | (5) |
| $\mathbf{u}_{\mathbf{s}}\left(\mathbf{x},t\right)\approx\sum_{k=1}^{N_{s}} \mathbf{U}_{k}^{s}\left(t\right)N_{k}^{s}\left(\mathbf{x}\right),$ | (6) |

where $\mathbf{U}_{i}\left(t\right)$, $P_{j}\left(t\right)$, $\mathbf{U}_{k}^{s}\left(t\right)$ – the nodal values of each variable. Selecting appropriate shape functions is critical to ensure stability and accuracy. To prevent numerical instabilities such as pressure checkerboarding, the chosen velocity and pressure shape functions must satisfy the Ladyzhenskaya–Babuška–Brezzi (LBB) condition (Leborgne, 2023).

Substituting the finite element approximations into the weak forms and performing numerical integration (e.g., using Gaussian quadrature) over each element leads to the formation of local stiffness matrices, mass matrices, and force vectors. These local contributions are then assembled into global matrices and vectors, considering the connectivity of the elements. Boundary conditions, including the coupling conditions at $\partial\Omega_{fsi}$, are incorporated into the global system, possibly modifying the matrices and vectors to enforce the necessary constraints.

| $\boldsymbol{M}_{f}\frac{d\boldsymbol{U}_{f}}{dt}+\boldsymbol{K}_{f}\left( \boldsymbol{U}_{f} \right)\boldsymbol{U}_{f}+\boldsymbol{G}^{T}P=\mathbf{F},$ | (7) |
| --- | --- |
| $\mathbf{GU}_{f}=0,$ | (8) |
| $\mathbf{M}_{s}\frac{d^{2}\mathbf{U}_{s}}{dt^{2}}+\mathbf{K}_{s}(\mathbf{U}_{s})\mathbf{U}_{s}=\mathbf{F}_{s},$ | (9) |

where $\mathbf{M}$ – mass matrix, $\mathbf{K}$ – stiffness matrix, $\mathbf{G}$ – pressure gradient matrix, $\mathbf{F}$ – vector of external forces, including forces from the fluid interactions ($f$ – for fluid domain, *s* – for solid domain). Combining the discretized equations for fluid and solid into a single system:

| $\left[\begin{matrix} \mathbf{A}_{ff} & \mathbf{A}_{fp} & \mathbf{A}_{fs} \\ \mathbf{A}_{pf} & 0 & 0 \\ \mathbf{A}_{sf} & 0 & \mathbf{A}_{ss} \end{matrix}\right]\left[\begin{matrix} \mathbf{U}_{f} \\ P \\ \mathbf{U}_{s} \end{matrix}\right]=\left[\begin{matrix} \mathbf{F}_{f} \\ 0 \\ \mathbf{F}_{s} \end{matrix}\right],$ | (10) |
| --- | --- |

where $\mathbf{A}_{ff}$, $\mathbf{A}_{ss}$ – matrices for fluid and solid body, $\mathbf{A}_{fp}$, $\mathbf{A}_{pf}$ – velocity-pressure coupling matrices, $\mathbf{A}_{fs}$, $\mathbf{A}_{sf}$ – fluid structure interactions matrices. Equation (24) can be represented by:

| $\mathbf{AX}=\mathbf{B},$ | (11) |
| --- | --- |

where $\mathbf{A}$is the global coefficient matrix incorporating contributions from the fluid and solid domains, $\mathbf{X}$ is the vector of unknowns, including fluid velocities, pressures, and solid displacements at the nodes, and $\mathbf{B}$ is the global load vector.

To solve the nonlinear system, we employ the Newton–Raphson iterative method for $\mathbf{\Phi}\left(\mathbf{X}\right)=0$. We need to start with an initial guess $\mathbf{X}^{0}$, which can be obtained from the previous time step or an initial estimate:

| $\mathbf{R}^{k}=\mathbf{\Phi}\left(\mathbf{X}^{k}\right),$ | (12) |
| --- | --- |
| $\mathbf{J}^{k}=\left[\frac{\partial\Phi_{i}}{\partial X_{j}}\right]_{\mathbf{X}=\mathbf{X}^{k}},$ | (13) |
| $\mathbf{J}^{k}\Delta\mathbf{X}^{k}=-\mathbf{R}^{k},$ | (14) |
| $\mathbf{X}^{k+1}=\mathbf{X}^{k}+\Delta\mathbf{X}^{k}.$ | (15) |

Here we begin with residual evaluation, then the Jacobian matrix is constructed. Next linear system is solving, where $\Delta\mathbf{X}^{k}$ is the correction to the current solution estimate, and solution is updating. Convergence is assessed based on the norms of the residual and the solution update:

| $\delta\mathbf{X}^{k}=\frac{\left\Vert\Delta\mathbf{X}^{k}\right\Vert}{\left\Vert\mathbf{X}^{k+1}\right\Vert}\leq\varepsilon_{rel},$ | (16) |
| --- | --- |
| $\delta\mathbf{R}^{k}=\left\Vert\mathbf{R}^{k+1}\right\Vert\leq\varepsilon_{abs}.$ | (17) |

The tolerances $\varepsilon_{rel}$ and $\varepsilon_{abs}$are predefined based on the desired accuracy and problem specifics. Upon achieving convergence at the current time step $t^{n}$, the solution $\mathbf{X}^{n+1}$ is used as the initial guess for the next time step $t^{n+1}$. This process is repeated until the final simulation time is reached.


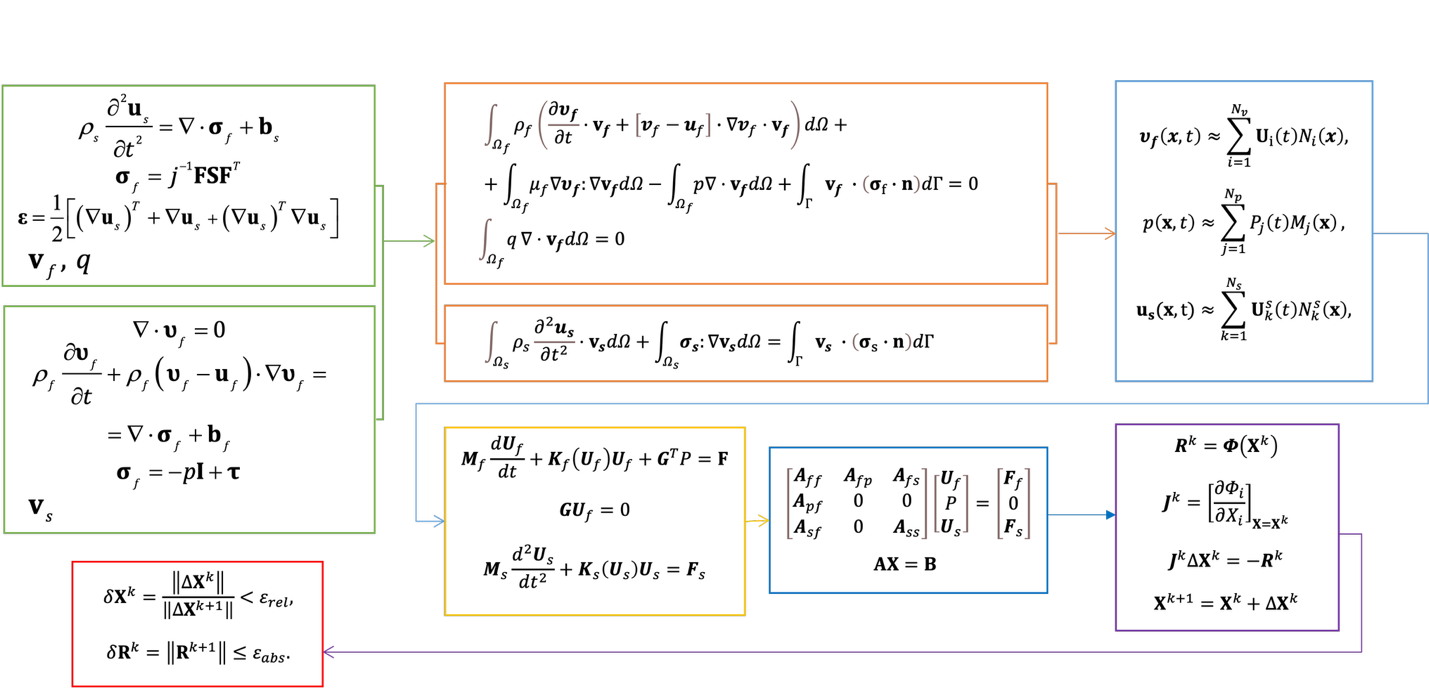


**Fig. B1.** Algorithm of FSI realisation in Comsol Multiphysics

**References:**

1. Averweg, S. *et al.* (2024) “A monolithic fluid–structure interaction approach using mixed LSFEM with high-order time integration,” *Computer Methods in Applied Mechanics and Engineering*, 423. Available at: https://doi.org/10.1016/j.cma.2024.116783.
2. Leborgne, G. (2023) “Inf-sup condition and locking: Understanding and circumventing. Stokes, Laplacian, bi-Laplacian, Kirchhoff--Love and Mindlin--Reissner locking type, boundary conditions.” Available at: http://arxiv.org/abs/2301.04373.
3. Zimbrod, P., Fleck, M. and Schilp, J. (2024) “An Application-Driven Method for Assembling Numerical Schemes for the Solution of Complex Multiphysics Problems,” *Applied System Innovation*, 7(3). Available at: https://doi.org/10.3390/asi7030035.
